# Development and initial validation of a modular life-course health education and science communication needs scale for Chinese clinical and community service users: a Tianjin-based study

**DOI:** 10.3389/fpubh.2026.1905218

**Published:** 2026-07-23

**Authors:** Dan Lv, Yunlu Zhang, Weilian Ni, Hongfei Xu, Yingxian Fang, Yuanyuan Wang, Xiaoping Wang

**Affiliations:** 1 Department of Critical Care Medicine, Tianjin Third Central Hospital, Tianjin, China; 2 The Third Central Clinical College of Tianjin Medical University, Tianjin, China; 3 Department of Orthopedics, Tianjin Third Central Hospital, Tianjin, China; 4 Department of Gynecology, Tianjin Third Central Hospital, Tianjin, China; 5 Department of Nursing, Tianjin Third Central Hospital, Tianjin, China

**Keywords:** community health services, health education, health equity, health literacy, life course, needs assessment, psychometric evaluation, science communication

## Abstract

**Purpose:**

This study aimed to develop and preliminarily validate a Modular Life-Course Health Education and Science Communication Needs Scale for Chinese clinical and community service users in Tianjin and their primary caregivers. The scale was intended as a modular needs-assessment instrument to identify perceived health education and science communication needs and communication preferences across life stages and caregiving contexts in local or comparable Chinese service settings, rather than as a tool already validated for national or international use.

**Methods:**

An initial item pool was developed through a systematic literature review, thematic analysis of semi-structured interviews (*n* = 22), two rounds of Delphi expert consultation (*n* = 15), and a pilot survey, guided by life-course theory and the socio-ecological model of health. Psychometric evaluation was conducted using two independent samples. In Stage 1, item analysis and exploratory factor analysis (EFA) were conducted using data from 487 participants. In Stage 2, confirmatory factor analysis (CFA) was performed using data from 328 participants. Internal consistency was assessed at the module level and, where applicable, at the subdimension level using Cronbach’s alpha coefficients with 95% confidence intervals, McDonald’s omega coefficients, Spearman–Brown corrected split-half reliabilities, and corrected item–total correlations. Content validity was evaluated using the content validity index (CVI). Convergent and discriminant validity were examined using average variance extracted (AVE) and the Fornell–Larcker criterion.

**Results:**

The final scale comprised 10 modules and 90 items, including six age-specific modules, two context-specific modules, and two preference modules. The age-specific and context-specific modules each encompassed three dimensions: physiological, psychological, and social needs. The response rates for both Delphi rounds were 100%. The expert authority coefficients (*Cr*) were 0.86 and 0.88, and Kendall’s coefficients of concordance (*W*) were 0.36 and 0.39, respectively (both *p* < 0.001). The item-level content validity index (I-CVI) ranged from 0.87 to 1.00, and the scale-level content validity index based on the averaging method (S-CVI/Ave) was 0.96. Item analysis showed that item-total correlation coefficients ranged from 0.52 to 0.89 (all *p* < 0.05), and critical ratio values ranged from 3.12 to 8.45 (all *p* < 0.01). EFA using principal axis factoring with Promax rotation supported three correlated factors for the age-specific and context-specific modules, corresponding to physiological, psychological, and social needs. Across these modules, the cumulative variance explained ranged from 63.86 to 75.44%, communalities ranged from 0.410 to 0.860, and primary pattern coefficients ranged from 0.601 to 0.900. A single factor was extracted for each preference module, explaining 61.31 and 59.47% of the variance for the format and channel preference modules, respectively; across the two preference modules, factor coefficients ranged from 0.732 to 0.808 and communalities ranged from 0.535 to 0.652. CFA provided preliminary evidence of acceptable model fit across modules, with *χ^2^*/*df* ranging from 1.28 to 2.41, RMSEA from 0.031 to 0.068, CFI from 0.95 to 0.99, TLI from 0.94 to 0.98, and SRMR from 0.028 to 0.055. AVE values for multi-item dimensions ranged from 0.54 to 0.74 across the age-specific and context-specific modules, and from 0.61 to 0.63 across the preference modules, providing preliminary evidence of convergent validity. The single-item social dimension of the hospice and palliative care module was interpreted descriptively rather than through AVE or CR. At the module level, Cronbach’s alpha coefficients ranged from 0.80 to 0.87, McDonald’s omega coefficients ranged from 0.81 to 0.88, Spearman–Brown corrected split-half reliability coefficients ranged from 0.77 to 0.84, and corrected item–total correlations ranged from 0.52 to 0.82. Subdimension-level reliability estimates were also acceptable.

**Conclusion:**

The Modular Life-Course Health Education and Science Communication Needs Scale showed acceptable preliminary psychometric performance in a Tianjin-based sample of Chinese clinical and community service users and their primary caregivers. The scale may support structured, stage-specific, and context-sensitive assessment of perceived health education and science communication needs and communication preferences in local or comparable Chinese service settings. However, the findings should be interpreted as geographically bounded, and further multicenter, cross-regional, rural–urban, and cross-cultural validation is required before broader national or international application.

## 1 Introduction

Promoting health literacy across the life course is central to the Healthy China 2030 initiative and to global public health improvement (1, 2). However, providing accurate, accessible, and contextually appropriate health information to heterogeneous populations remains challenging. A structured assessment of perceived health education and science communication needs is therefore necessary before designing population-oriented health literacy activities (3, 4). In this study, these needs refer to individuals’ or caregivers’ perceived need for understandable, trustworthy, and contextually relevant educational content that supports health understanding, caregiving, self-management, health information appraisal, and linkage to appropriate services. These perceived content needs are distinct from communication preferences, which refer to preferred formats and channels for receiving health information. Guided by life-course theory and the socio-ecological model of health, this study developed and initially validated a modular needs-assessment scale for Chinese clinical and community service users and their primary caregivers.

Life course theory, originally proposed by the sociologist Glen H. Elder, provides a foundational temporal lens for understanding the dynamic evolution of human health needs (5). A core tenet of this theory is that health risks, health awareness, and preferences for accessing health information change systematically across developmental stages and social transitions. Health concerns that are highly salient during adolescence, such as reproductive health, differ markedly from those prioritized by older adults, such as fall prevention. This stage-specific heterogeneity renders generic assessment tools insufficient for capturing the nuanced health information needs of different populations across the life course. This perspective is also consistent with the life course orientation emphasized in the Healthy China 2030 Planning Outline, which extends health promotion from fetal development to the end of life (1). Accordingly, this instrument divides the continuous life course into six age-specific modules: infancy and toddlerhood, preschool age, school age, adolescence, adulthood, and older adulthood. Each module is tailored to reflect the developmental characteristics and stage-specific health risks of its intended population.

Life course theory further emphasizes that certain critical life transitions, such as childbearing and end-of-life care, may occur across different age groups. The health-related tasks and information needs triggered by these transitions are context-specific rather than solely age-dependent, and therefore cannot be fully captured by a single age-based module (5, 6). Therefore, the proposed scale includes two independent context-specific modules: the pregnancy and maternity module and the hospice and palliative care module. Notably, these critical transitions often intersect with structural vulnerabilities. For example, individuals with lower socioeconomic status may face disproportionate barriers to accessing reliable health information during pregnancy and maternity, whereas patients receiving hospice and palliative care may have limited access to online resources because of disease burden, caregiver dependence, or the digital divide. The inclusion of independent context-specific modules facilitates the identification of, and targeted response to, differentiated needs that may be amplified by structural inequalities.

Although life course theory addresses the temporal continuity of health needs, it does not fully account for the multilevel contextual factors that shape individuals’ access to health information. To address this limitation, the present study integrates the socio-ecological model of health (7), which posits that individual health is influenced by environmental factors spanning family, community, institutional, and policy contexts (8). For instance, caregivers’ nutritional knowledge and feeding practices during infancy and toddlerhood are shaped not only by child development but also by family support and the accessibility of community services (9, 10). Similarly, chronic disease management among older adults is closely linked to the distribution of family caregiving responsibilities and the continuity of primary care services (11, 12). Existing instruments have largely neglected these social determinants (7). Therefore, each age-specific and context-specific module in the proposed scale encompasses three dimensions—physiological, psychological, and social needs—with particular attention to structural factors such as family support, community resources, and barriers to digital participation (13).

Even when age-specific health content is precisely identified, the anticipated public health impact may remain unrealized if the information is delivered through channels or formats that do not align with audience preferences. The Chinese Expert Consensus on the Life-Course Active Health Science Communication Framework systematically categorizes public health communication needs into three core and interrelated dimensions: content needs, referring to what information audiences require; channel needs, referring to the preferred channels for receiving information; and format needs, referring to the forms in which audiences can best absorb and understand information (14). This framework is particularly important for guiding effective health education and science communication, as mounting empirical evidence shows that the alignment between communication strategies and audience preferences is closely associated with the reach, acceptability, and potential behavioral impact of health information (15, 16). For example, although short-video platforms have become a dominant channel for health information among the general population, older adults often prefer in-person health lectures delivered by community physicians or traditional media (17), whereas pregnant and postpartum women tend to prioritize official hospital websites or prenatal education programs (18). Guided by this health communication needs assessment framework, the proposed scale includes two dedicated preference modules: the health science communication format preference module and the health science communication channel preference module (hereafter referred to as the format preference module and the channel preference module, respectively). These two modules were designed as brief pragmatic preference-profile tools rather than as conventional latent trait scales. The format module identifies respondents’ preferred forms of health education and science communication, whereas the channel module identifies preferred access routes. Because communication formats and channels are not mutually exclusive in real-world community health practice, the primary interpretation of these modules should focus on item-level profiles and priority rankings rather than on total scores alone. The module mean score may be used only as a broad indicator of overall receptivity to multiple communication options.

## 2 Materials and methods

### 2.1 Development of the initial scale

#### 2.1.1 Conceptual and operational definition of the construct

In the present study, “health education and science communication needs” were defined as the perceived need of individuals or their primary caregivers for health-related educational and science communication content that is understandable, trustworthy, life-stage appropriate, and relevant to their health tasks, caregiving roles, psychosocial concerns, and service contexts. Operationally, a health education and science communication need was considered present when respondents indicated that they required information, explanation, guidance, or resource linkage related to physiological health, psychological adaptation, or social support that could reasonably be addressed through health education or science communication activities.

This construct was distinguished from several related but different concepts. First, it referred to perceived needs rather than objectively assessed educational deficits. The scale did not include a knowledge test, clinical assessment, professional diagnosis, or external evaluation of whether respondents lacked specific health knowledge. Therefore, higher scores indicate greater perceived salience or priority of health education and science communication content from the respondent’s perspective, rather than objectively verified knowledge deficits. Second, health education and science communication needs were distinguished from health concerns or general interest. A health concern or interest was considered relevant to the construct only when it implied a perceived requirement for educational content, explanation, decision support, self-management guidance, or access to reliable resources. Third, the construct was distinguished from readiness to receive information; the present scale assessed what content respondents perceived as needed, but it did not directly measure motivation, intention, or behavioral readiness to participate in health education activities.

The age-specific and context-specific modules were designed to measure perceived content needs. These modules covered physiological, psychological, and social domains, reflecting the life course and socio-ecological orientation of the scale. By contrast, the two preference modules were designed to assess communication preferences rather than content needs. The format preference module assessed preferred forms of receiving health education and science communication, whereas the channel preference module assessed preferred access routes or sources. Thus, the preference modules were intended to inform how and through which routes health information should be delivered, whereas the age-specific and context-specific modules were intended to identify what content respondents perceived as needed.

#### 2.1.2 Literature review

A systematic search was conducted across Chinese and English databases, including PubMed, Web of Science, Cochrane Library, Embase, CINAHL, China National Knowledge Infrastructure (CNKI), Wanfang Data, and the VIP Database. The Chinese search terms included “health science communication,” “health education,” “health literacy,” “health communication,” “life course,” “life stage,” “health needs,” “health information needs,” “health promotion,” “scale development,” “reliability,” and “validity.” The English search terms included “health literacy,” “health education,” “health communication,” “science communication,” “life course,” “life cycle,” “health information needs,” “health promotion,” “scale development,” “reliability,” and “validity.” The search covered the period from database inception to January 31, 2026.

The literature screening focused on four key domains: (1) policies, guidelines, expert consensus statements, and theoretical studies related to health education, health science communication, and health communication; (2) studies examining health information needs and health education and science communication needs among populations at different life stages; (3) health education and science communication tools or needs assessment instruments related to children, adolescents, adults, older adults, pregnancy and maternity, and hospice and palliative care; and (4) methodological literature on scale development, cross-cultural adaptation, and psychometric evaluation. Based on the literature review, the research team developed a preliminary conceptual framework and initial item pool for the proposed scale (19).

#### 2.1.3 Semi-structured interviews

Using purposive sampling, individuals at different life stages or their primary caregivers were recruited from a tertiary hospital and community health service centers in Tianjin between February 1 and February 18, 2026, for semi-structured interviews. Recruitment aimed to obtain variation across life stage, respondent type, and service setting, and to ensure that each age-specific and context-specific module was informed by relevant service-user or caregiver perspectives. The inclusion criteria were as follows: (1) aged 18 years or older, or serving as the primary caregiver for individuals in infancy and toddlerhood, preschool age, school age, or adolescence (caregivers aged ≥18 years); (2) clear consciousness, ability to communicate effectively in Mandarin or the Tianjin dialect, and capacity to understand the interview guide and express personal experiences and opinions; and (3) provision of informed consent and voluntary participation. The exclusion criteria were as follows: (1) severe hearing or language impairment precluding in-depth oral communication; (2) previous or current diagnosis of mental illness or overt cognitive impairment; (3) pregnancy requiring strict bed rest; and (4) experience of a major negative life event within the preceding month, such as bereavement or a major natural disaster, resulting in severe acute stress. The interviews were conducted by researchers who had received standardized training. Each interview lasted approximately 20–30 min and was audio recorded with the participant’s consent.

The interview guide combined a common set of open-ended questions with module-specific probes. Core questions explored the health topics participants most wanted to understand, difficulties in obtaining, judging, and applying health information, perceived physiological, psychological, and social support needs, caregiving or family-role challenges, and preferred formats and channels for receiving health education and science communication. The guide was then tailored to each life stage. For infancy and toddlerhood and preschool age, the interviews focused on feeding and caregiving, safety protection, early development, kindergarten adaptation, and family support. For school age and adolescence, the interviews focused on academic stress, mental health, digital health literacy, sexuality education, peer relationships, and parent–child communication. For adulthood, the interviews focused on occupational health, early screening for chronic diseases, work–family balance, health information appraisal, and social support. For older adulthood, the interviews focused on chronic disease management, fall prevention, rational medication use, cognitive maintenance, retirement adjustment, digital inclusion, and eldercare resources. For pregnancy and maternity, the interviews focused on prenatal care, childbirth preparation, postpartum recovery, newborn care, the distribution of family caregiving responsibilities, and protection of workplace rights. For hospice and palliative care, the interviews focused on symptom management, comfort care, psychological support, expression of care preferences, advance care planning, family support, and linkage to relevant resources.

Following the principle of information saturation (20), a total of 22 participants were interviewed, including three caregivers of infants and toddlers, two caregivers of preschool children, three school-age child–caregiver dyads, two adolescent–caregiver dyads, three adults, three older adults, three individuals in the pregnancy and maternity period, and three caregivers of individuals receiving hospice and palliative care. Among them, eight were male and 14 were female, with an age range of 18–76 years and a mean age of 44.3 ± 15.6 years. Saturation was assessed concurrently during data collection and preliminary analysis. Recruitment stopped after all major life-stage and context-specific groups had been represented and the final interviews no longer generated new major themes or item concepts within the predefined life course and socio-ecological framework. Audio recordings were transcribed verbatim, anonymized, and checked against the original recordings before analysis. Thematic analysis was conducted by two trained researchers, who first read all transcripts repeatedly and then independently performed open coding. Similar codes were grouped into categories and then into higher-order themes. Coding disagreements were resolved through discussion, and a senior researcher was consulted when consensus could not initially be reached. Coding and theme matrices were managed manually using Microsoft Word and Excel. The qualitative analysis team included researchers with backgrounds in clinical medicine, nursing, public health, health education and science communication, and psychology, which enabled clinical, community, psychosocial, and communication-related interpretations to be compared during theme development. To strengthen reflexivity, the interviewers used neutral prompts, avoided steering participants toward provider-defined priorities, kept reflexive notes after interviews, and compared emerging codes with participants’ own wording. Five key themes were identified: (1) barriers to knowledge acquisition; (2) insufficient content tailoring; (3) psychological and cognitive factors; (4) influence of social support systems; and (5) limitations of service delivery channels. Additional details on recruitment, coding procedures, coder backgrounds, saturation, reflexivity, and illustrative quotations are provided in Supplementary Table 7.

#### 2.1.4 Generation of the item pool

The research team comprised members with backgrounds in clinical medicine, nursing, public health, health education and science communication, and psychology. Based on the theoretical framework, literature review, and interview findings, the initial item pool was developed following the logic of “life stage-health task-health education and science communication need–measurable expression.” Consistent with recommended procedures for scale development and COSMIN-informed principles for content validity (21, 22), item development adhered to five principles: (1) each item should express only one core perceived content need; (2) items should use language understandable to the general public while avoiding excessive technical terminology; (3) item wording should focus on needs that could be addressed through health education or science communication, rather than clinical treatment procedures, diagnostic decisions, or objective knowledge testing; (4) age-specific and context-specific modules should cover physiological, psychological, and social needs; and (5) the number of items should balance content coverage with response burden. The initial item pool comprised 94 items, covering infancy and toddlerhood, preschool age, school age, adolescence, the working period, which was subsequently standardized as adulthood, pregnancy and maternity, older adulthood, hospice and palliative care, and preferences for health science communication formats and channels. Participant input was explicitly used to convert qualitative themes into candidate item concepts (36). For example, participants’ descriptions of contradictory online information and difficulty understanding professional terminology informed items on reliable information sources, health information appraisal, and plain-language explanations. Accounts of examination stress, pregnancy-related anxiety, fear of falling, and caregiver guilt supported the inclusion of psychological-need items across adolescence, pregnancy and maternity, older adulthood, and hospice and palliative care modules. Comments about school–family collaboration, intergenerational caregiving conflict, social isolation, and limited access to service resources informed social-need items, whereas comments about inconvenient offline lectures, distrust of online information, and the need for individualized consultation informed the format and channel preference modules. Supplementary Table 7 presents the qualitative audit trail, including illustrative quotations and their links to item generation.

#### 2.1.5 Delphi expert consultation

##### 2.1.5.1 Expert selection

Experts were eligible if they met the following criteria: (1) bachelor’s degree or higher; (2) an intermediate or higher professional title; (3) at least 5 years of clinical, teaching, research, or management experience in pediatrics, obstetrics and gynecology, geriatrics, hospice and palliative care, public health, nursing, health education and science communication, psychology, or related fields; (4) familiarity with life course health management, health science communication, or scale development; and (5) willingness to participate in the study. To ensure that all modules were reviewed by experts with relevant content expertise, the Delphi panel was constructed to cover the main life-stage, clinical, community, psychosocial, and communication domains represented in the scale. The panel included experts in general practice, pediatrics, obstetrics and gynecology, geriatrics, hospice and palliative care, nursing management, psychology, public health, and health education and science communication. Module-specific expertise was considered during expert invitation: pediatric and child-health experts reviewed the infancy and toddlerhood, preschool-age, school-age, and adolescence modules; obstetrics and gynecology experts reviewed the pregnancy and maternity module; geriatrics, general practice, nursing, and hospice and palliative care experts reviewed the older adulthood and hospice and palliative care modules; and experts in public health, health education and science communication, and psychology reviewed the overall conceptual framework, psychological and social dimensions, and the format and channel preference modules.

##### 2.1.5.2 Development of the consultation questionnaire and indicator screening

Two rounds of Delphi expert consultation were conducted from February 25 to March 25, 2026 (23). The consultation questionnaire comprised an invitation letter to experts, a scale conceptual framework evaluation form, an item importance evaluation form, an expert authority self-assessment form, and an expert demographic information form. Experts were invited to evaluate the appropriateness of module classification, item importance, wording clarity, content comprehensiveness, accuracy of target respondents, and presence of duplicate or missing items. Item importance was evaluated using a 5-point Likert scale, with 1 indicating “not important at all” and 5 indicating “very important.” Expert authority was determined on the basis of the familiarity coefficient (Cs) and the judgment basis coefficient (Ca), with the authority coefficient computed as Cr = (Cs + Ca)/2.

Following recommended Delphi reporting principles, item retention and revision decisions were made according to both quantitative ratings and qualitative expert comments. Items were retained when they had acceptable importance ratings, low dispersion of expert opinion, and clear relevance to the conceptual definition of perceived health education and science communication needs. Items were revised when experts considered the content relevant but suggested improvements in wording clarity, target respondent accuracy, dimensional attribution, or consistency with public-oriented health education language. Items were merged when two or more items overlapped conceptually or addressed closely related needs within the same module and dimension. Items were deleted when they were judged to be redundant, outside the construct definition, too close to clinical diagnosis or treatment procedures, insufficiently applicable to the target population, or likely to increase respondent burden without adding distinct content coverage. Final item decisions were made by the research team after discussion of expert ratings, written comments, the theoretical framework, and the modular structure of the scale.

### 2.2 Pilot survey

A pilot survey was conducted among 20 community residents from March 28 to April 5, 2026, with two to three participants recruited from each life stage or health context. The recruitment sources and inclusion and exclusion criteria were consistent with those for the semi-structured interviews. The pilot survey was conducted to evaluate the clarity of item wording, feasibility of the modular administration process, applicability of response versions, and appropriateness of the physiological-psychological-social three-dimensional structure. The findings indicated that participants were able to understand the items and complete the questionnaire without difficulty. Accordingly, the terminology and selected item wording were further standardized and refined.

### 2.3 Formal scale testing

#### 2.3.1 Participants

Convenience sampling was used to recruit participants for the formal survey from April 8 to May 20, 2026, from Tianjin-based healthcare institutions, community health service centers, eldercare facilities, and hospice/palliative care settings. The core inclusion and exclusion criteria were adapted from those used in the semi-structured interview stage and applied according to the respondent’s life stage, caregiving role, and ability to complete the questionnaire independently or with investigator assistance. For exploratory factor analysis, the recommended sample size is generally 5–10 times the number of items (24, 25). For confirmatory factor analysis, although a total validation sample of 200 or more participants is often considered desirable in conventional scale validation studies (26), this criterion could not be directly applied to the present study because the proposed scale used a modular administration design and each module was completed by a different eligible respondent subgroup. Therefore, the relevant denominator for each CFA model was the module-specific CFA-stage sample size rather than the total Stage 2 sample. Given the preliminary nature of this initial validation study and the practical difficulty of recruiting sufficient respondents for some life-stage and context-specific modules, especially hospice and palliative care, the CFA results were interpreted as preliminary model-fit evidence rather than definitive confirmatory validation. In the present initial validation study, the target population was defined as Chinese clinical and community service users and their primary caregivers recruited from Tianjin-based healthcare, community health, eldercare, and hospice/palliative care settings. Therefore, the findings should be interpreted as preliminary evidence generated from a geographically bounded service-user sample rather than as evidence of national or international generalizability. To address the modular validation design, demographic and contextual characteristics were summarized separately for the EFA-stage and CFA-stage samples and further stratified by module. These characteristics included module-specific sample size, target-person age range or description, respondent type, respondent age, sex, educational attainment, marital status, self-rated health status, and survey setting.

#### 2.3.2 Survey instruments

The formal questionnaire consisted of two sections. The first section was a general information questionnaire, including age, sex, marital status, educational attainment, occupational status, survey setting, life stage, health status, and habits of accessing health science communication information. The second section was the Modular Life-Course Health Education and Science Communication Needs Scale developed in this study. Respondents selected one age-specific module according to their own age or the age of the person under their care: infancy and toddlerhood (<3 years), preschool age (3 to <7 years), school age (7 to <10 years), adolescence (10 to <18 years), adulthood (18 to <60 years), or older adulthood (≥60 years). The adulthood module was treated as a broad adult module in this initial scale validation study. It was designed to assess health education and science communication needs that are commonly relevant across adult life before older adulthood, such as occupational health, prevention and early screening of chronic diseases, health information appraisal, mental health, social support, and family role adaptation. The present study did not further divide adulthood into early adulthood and middle adulthood modules; therefore, subgroup-specific psychometric performance within the adult age range was not evaluated. Individuals currently in the preconception period, pregnancy, the intrapartum period, or within 1 year postpartum additionally completed the pregnancy and maternity module. Individuals receiving hospice and palliative care, or their primary caregivers, additionally completed the hospice and palliative care module. All respondents additionally completed two preference modules: the health science communication format preference module and the health science communication channel preference module.

The specific structure of the proposed scale, number of items in each module, and response versions are presented in Table 1. The proposed scale used a 5-point Likert scoring method, with 1 indicating “no need at all,” 2 indicating “low need,” 3 indicating “moderate need,” 4 indicating “high need,” and 5 indicating “very high need.” For age-specific and context-specific modules, higher scores indicated greater perceived need for the corresponding health education and science communication content; these scores should not be interpreted as objective evidence of educational deficits or inadequate health literacy. Module total scores, module mean scores, and dimension mean scores were calculated for these content-need modules. For the preference modules, higher scores indicated stronger preference or receptivity toward the corresponding communication format or channel, rather than greater need for content. These modules were interpreted differently from the age-specific and context-specific content-need modules. They were not designed to classify respondents into mutually exclusive preference types or to rely primarily on a total score from a presumed latent construct. Instead, they were designed as pragmatic preference-profile modules. In practical use, individual item scores and rank-ordered preference profiles should be prioritized to identify the most acceptable formats and channels for specific populations. The module mean score was retained only as a supplementary summary indicator of overall receptivity to multiple communication options. Because respondents completed different age-specific and context-specific modules according to their life stage or health context, an overall score across all 90 items was not calculated.

**Table 1:** Structure, target respondents, and administration versions of the modular life-course health education and science communication needs scale.

Module type	Module name	Target respondents	No. of items	No. of dimensions	Response version
Age-specific module	Infancy and toddlerhood (Y)	Caregivers of children aged <3 years	10	3	Caregiver-report version
Age-specific module	Preschool age (X)	Caregivers of children aged 3 to <7 years	10	3	Caregiver-report version
Age-specific module	School Age (L)	Children aged 7 to <10 years and their caregivers	10	3	Hybrid version
Age-specific module	Adolescence (Q)	Adolescents aged 10 to <18 years and their caregivers	10	3	Self-report/caregiver-report version
Age-specific module	Adulthood (Z)	Adults aged 18 to <60 years	10	3	Self-report version
Age-specific module	Older adulthood (S)	Older adults aged ≥60 years	10	3	Self-report version
Context-specific module	Pregnancy and maternity (C)	Individuals from preconception to 1 year postpartum	12	3	Self-report/caregiver-report version
Context-specific module	Hospice and palliative care (A)	Individuals receiving hospice and palliative care and their caregivers	8	3	Self-report/caregiver-report version
Preference module	Format preference (F)	All respondents	5	1	Self-report/caregiver-report version
Preference module	Channel preference (D)	All respondents	5	1	Self-report/caregiver-report version
Total	10 modules	—	90	—	—

The caregiver-report version indicates that the module was completed by the primary caregiver as a proxy respondent. The hybrid version used in the school-age module indicates a joint child–caregiver assisted completion process, in which the child was encouraged to express his or her perceived needs while the caregiver provided assistance with comprehension and contextual confirmation when necessary. This process generated one response set rather than two independent parallel ratings from the child and caregiver. The self-report/caregiver-report version indicates that the questionnaire may be completed by the respondent or by the caregiver as a proxy respondent according to the respondent’s age, cognitive status, health condition, and actual ability to complete the questionnaire. Module codes are used for item numbering; for example, Y1 refers to the first item in the infancy and toddlerhood module. Age-specific and context-specific modules each include three subdimensions: physiological, psychological, and social needs. Preference modules were specified as unidimensional brief preference indices. Their module mean scores indicate overall receptivity to different communication formats or channels, whereas individual item scores may be used to identify specific preferred formats or access routes.

#### 2.3.3 Data collection and quality control

Before the formal survey, all investigators received standardized training covering the study objectives, inclusion and exclusion criteria, informed consent procedures, module selection rules, questionnaire completion procedures, responses to frequently asked questions, and data confidentiality requirements. Using standardized instructions, investigators explained the purpose and significance of the study to respondents. After providing informed consent, respondents completed the questionnaire anonymously. Respondents who were able to read independently completed the questionnaire by themselves. For respondents with reading difficulties or poor vision, investigators read each item and response option aloud, and recorded the questionnaire according to the respondent’s verbal answers. The response and administration modes were determined according to the target respondent’s developmental stage, cognitive and physical condition, caregiving context, and ability to complete the questionnaire independently. These modes included self-report, caregiver proxy report, joint child–caregiver assisted completion, and investigator-administered completion for respondents with reading difficulties or poor vision. Because response mode was not randomly assigned and was partly determined by life stage and health condition, the present study was not designed to establish psychometric equivalence across response or administration modes. Therefore, the findings should be interpreted as preliminary evidence for the modular scale under its intended pragmatic administration conditions, rather than as evidence that self-report, caregiver-report, hybrid, and investigator-administered responses are fully interchangeable. Upon completion, completed questionnaires were reviewed on site for missing responses, obvious logical errors, and incorrect module selection.

Double data entry and independent verification were performed by two researchers. Questionnaires with missing values, outliers, or inconsistencies with the module selection rules were examined. Questionnaires were considered invalid if key variables were missing, if the module selection rules were inconsistent with the respondent’s life stage or health context, or if more than one item was missing within a completed module. For age-specific and context-specific modules, if no more than one item was missing within a completed module, the missing value was imputed using the mean of the completed items within the corresponding dimension. For the two preference modules, if no more than one item was missing, the missing value was imputed using the module mean score. If more than one item was missing within a completed preference module, the corresponding module response was excluded from module-level analysis. Item-level missing data rates and module-specific missing data handling procedures are reported in Supplementary Table 3. The Stage 1 sample was used for item analysis and exploratory factor analysis, whereas the Stage 2 sample was used for confirmatory factor analysis. The two samples were mutually independent to reduce the risk of model overfitting caused by using the same sample for both exploration and validation. The detailed sample flow, including the numbers of individuals approached, screened, enrolled, excluded, and included in the final analytic samples, as well as the reasons for invalid questionnaires, is reported in Supplementary Table 5.

#### 2.3.4 Statistical analysis

To evaluate whether the independent EFA-stage and CFA-stage samples were comparable, key demographic and contextual characteristics were compared between the two validation stages. Respondent age was compared using an independent-samples t test. Categorical variables, including sex, educational attainment, self-rated health status, survey setting, age-specific module membership, and completion of context-specific modules, were compared using Pearson’s chi-square test. Because the pregnancy and maternity module and the hospice and palliative care module were context-specific modules that were not mutually exclusive with age-specific module membership, their completion status was compared separately as binary variables. The two preference modules were completed by all respondents and were therefore not statistically tested. These analyses were conducted descriptively to assess between-stage comparability, and a two-sided *p* value <0.05 was considered statistically significant.

##### 2.3.4.1 Item analysis

All psychometric analyses were conducted independently for each module. Item screening was performed using the item-total correlation method, critical ratio method, and Cronbach’s alpha coefficient method. First, for the item-total correlation method, Pearson correlation coefficients were calculated between each item score and the corresponding module total score. Items with a correlation coefficient <0.40 or *p* > 0.05 were considered for deletion. Second, for the critical ratio method, the top 27% of respondents according to module total scores were assigned to the high-score group, and the bottom 27% were assigned to the low-score group. Differences in item scores between the two groups were compared, and items with a critical ratio <3.0 or *p* > 0.05 were considered for deletion. Third, for the Cronbach’s alpha coefficient method, if deletion of an item increased the module’s Cronbach’s *α* coefficient by >0.05, the item was considered for deletion. Final decisions regarding item retention or deletion were made by comprehensively considering the theoretical framework and expert opinions.

##### 2.3.4.2 Validity testing

###### 2.3.4.2.1 Content validity

Content validity was evaluated using expert ratings from the Delphi consultation. In the Delphi questionnaire, experts provided a 5-point quantitative rating for item importance, which reflected the relevance and necessity of each item for assessing perceived health education and science communication needs. Other aspects, including wording clarity, content comprehensiveness, target-respondent appropriateness, duplicate content, and missing content, were evaluated through written expert comments and were used for item revision rather than for the numerical CVI calculation. The item-level content validity index (I-CVI) was calculated as the proportion of experts who rated an item as 4 or 5 on the item-importance scale. The scale-level content validity index based on the averaging method (S-CVI/Ave) was calculated as the mean I-CVI across all retained items. To correct for chance agreement among experts, the modified kappa statistic was also calculated using the formula k* = (I-CVI − Pc)/(1 − Pc), where Pc = [N!/(A!(N − A)!)] × 0.5 N, N is the number of experts, and A is the number of experts rating the item as 4 or 5. An I-CVI ≥ 0.78, S-CVI/Ave ≥ 0.90, and modified kappa ≥0.74 were considered to indicate satisfactory content validity.

###### 2.3.4.2.2 Construct validity

Exploratory factor analysis (EFA) was conducted independently for each module. Because the purpose of EFA was to examine latent constructs rather than merely reduce observed variables, principal axis factoring was used as the extraction method, in line with methodological recommendations favoring common factor analysis over principal component analysis for latent construct validation (27). For age-specific and context-specific modules, Promax oblique rotation was applied because the physiological, psychological, and social dimensions were theoretically expected to be correlated. For the two preference modules, no rotation was applied when a single-factor solution was extracted. Factor retention was determined using a combined decision framework rather than relying solely on the eigenvalue >1 rule. Specifically, Horn’s parallel analysis, scree plot inspection, eigenvalues >1, theoretical interpretability, consistency with the predefined modular framework, cross-loading diagnostics, and residual correlation diagnostics were considered together (28). In parallel analysis, factors were retained when the observed eigenvalue exceeded the corresponding eigenvalue generated from random data with the same sample size and number of items. Scree plots were inspected to identify the point of inflection. Items were evaluated using primary pattern coefficients, communalities, cross-loadings, and theoretical attribution. A primary pattern coefficient ≥0.40 was considered acceptable. Items were flagged for further review if they showed substantial cross-loading, defined as a secondary pattern coefficient at or above 0.30 or a primary-secondary difference less than 0.20. Communalities below 0.40 were flagged for review rather than used as an automatic deletion criterion, because item retention also considered conceptual coverage and expert judgment. Residual diagnostics were examined using residual correlations from the reproduced correlation matrix to identify large or systematically clustered residual correlations suggesting the need for additional factors. Factor assignment was based on the pattern matrix rather than the structure matrix. The factor-retention evidence for each module is summarized in Supplementary Table 8.

CFA was conducted independently for each module using the independent Stage 2 sample, with the module-specific CFA-stage sample size as the denominator; the two preference modules used the full Stage 2 sample because all respondents completed them. AMOS version 26.0 was used. Because the items used five ordered Likert-type response categories, maximum likelihood (ML) estimation in AMOS 26.0 was retained as a pragmatic estimator rather than as an assumption-free estimator. Module-specific skewness, kurtosis, and Mardia’s multivariate kurtosis were inspected to evaluate distributional conditions before interpreting the CFA results. As shown in Supplementary Table 10, severe univariate non-normality was not observed; however, evidence of multivariate non-normality was present. Robust ML estimation, ordinal-data estimators such as WLSMV/DWLS, and sensitivity analyses using alternative estimators were not performed in the present study. Therefore, standard errors, chi-square statistics, and model-fit indices were interpreted cautiously, particularly for modules with small CFA-stage samples. Three-dimensional correlated-factor CFA models were specified for the age-specific modules and the pregnancy and maternity module. For the hospice and palliative care module, the physiological and psychological domains were estimated as multi-item latent factors. However, the social domain was represented by only one item, A8, concerning family caregiving support, grief counseling, and linkage to resources. To avoid identifying a single-indicator latent factor through unverifiable assumptions, A8 was not specified as a fully estimated latent social factor in the CFA. No factor loading, residual variance, reliability coefficient, or other parameter was fixed to identify a single-indicator social factor. Instead, A8 was retained as an observed descriptive indicator and for module-level scoring, whereas latent-variable indices such as AVE, CR, discriminant validity, and subdimension-level reliability were not calculated for this single-item social domain. Standardized factor loadings, latent factor correlations, residual correlations, and model modifications were examined and reported. Model fit was evaluated using *χ*^2^/df, RMSEA, CFI, TLI, IFI, and SRMR (29). Convergent validity was assessed using AVE, and discriminant validity using the Fornell–Larcker criterion. The CFA subsamples were 50, 40, 45, 37, 80, 76, 45, and 28 for the eight age-specific or context-specific modules, respectively; therefore, these CFA results were interpreted as preliminary rather than definitive.

###### 2.3.4.2.3 Criterion-related validity

Criterion-related validity was not evaluated in the present study because no external validated measure of health literacy, health education needs, digital health literacy, health information-seeking behavior, patient activation, or health-related behavioral outcomes was administered concurrently with the proposed scale. Therefore, the validity evidence in this study was limited to content validity, construct validity, convergent validity, and discriminant validity.

##### 2.3.4.3 Reliability testing

Internal consistency reliability was assessed at both the module and subdimension levels using the combined formal survey sample across the EFA and CFA stages. Because the proposed scale used a modular administration design, different age-specific and context-specific modules were completed by different eligible respondent groups, and all 90 items were not administered to the same respondents. Therefore, total-scale reliability across all items was not calculated. At the module level, Cronbach’s alpha coefficients with 95% confidence intervals, McDonald’s omega coefficients, Spearman–Brown corrected split-half reliability coefficients, and corrected item–total correlations were calculated separately for each module. At the subdimension level, Cronbach’s alpha coefficients with 95% confidence intervals, McDonald’s omega coefficients, and corrected item–subdimension-total correlations were calculated for the physiological, psychological, and social subdimensions of the age-specific and context-specific modules. The two preference modules were not included in the subdimension-level analysis because they were designed as unidimensional preference indices. Cronbach’s alpha and McDonald’s omega values greater than 0.70 were considered to indicate acceptable internal consistency. For two-item subdimensions, reliability estimates were interpreted cautiously, and the corrected item–subdimension-total correlation was considered equivalent to the inter-item correlation.

Test–retest reliability was not assessed in the present study because the formal psychometric testing used a cross-sectional design and did not include repeated administration of the scale at a predefined interval. Therefore, the reliability evidence reported in this study should be interpreted as evidence of internal consistency rather than temporal stability. Future studies should evaluate test–retest reliability for each module across appropriate retest intervals and respondent groups before the scale is used for longitudinal monitoring, repeated needs assessment, or evaluation of changes following health education interventions.

### 2.4 Research flowchart

**Figure 1:**
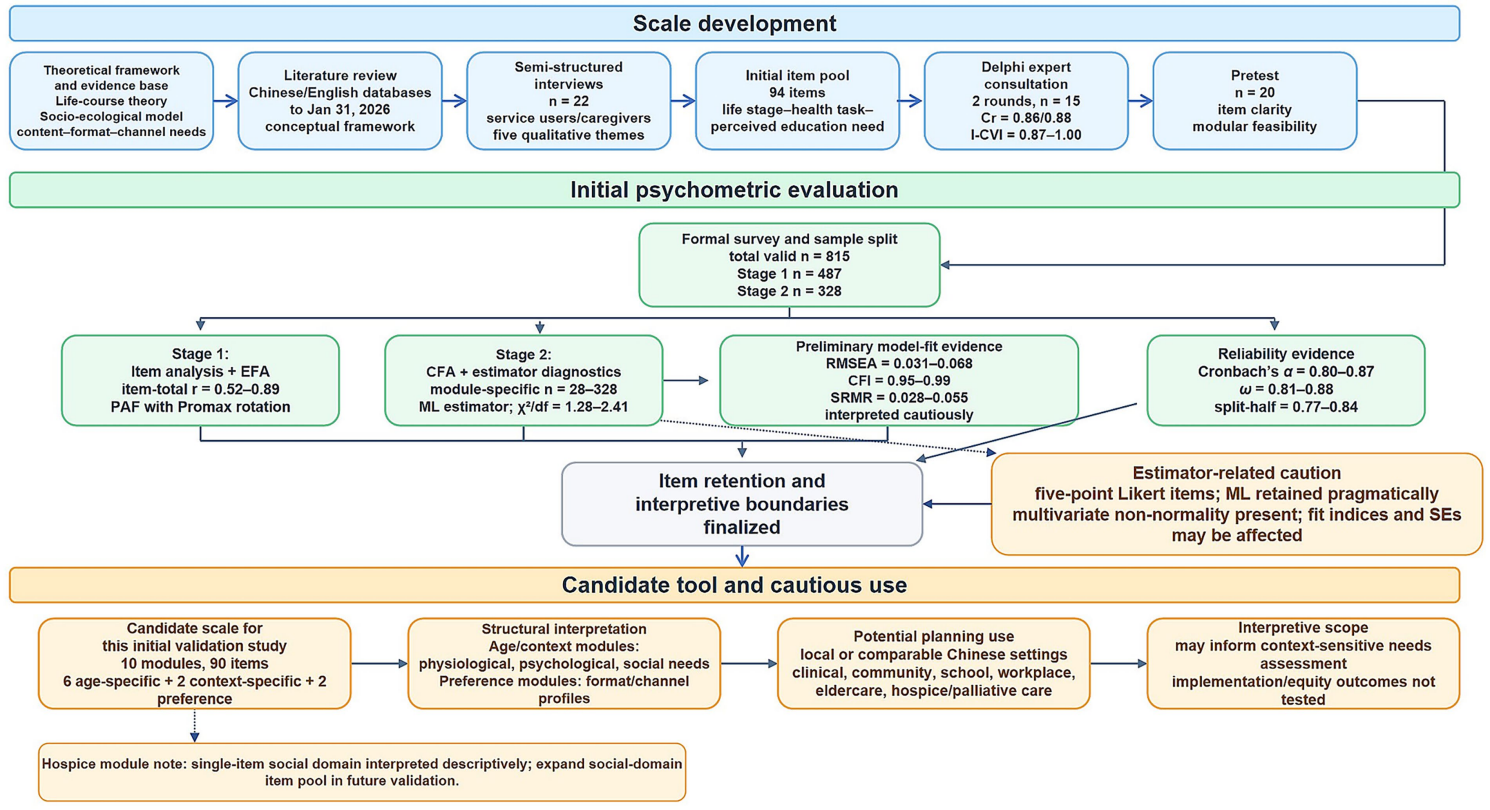
Development and initial validation workflow of the modular life-course health education and science communication needs scale. EFA, exploratory factor analysis; CFA, confirmatory factor analysis.

The overall workflow for scale development and initial psychometric validation is presented in Figure 1. The figure summarizes the sequential process of conceptual framework development, literature review, semi-structured interviews, item pool generation, Delphi expert consultation, pilot testing, and formal psychometric testing. It also indicates the formation of the final scale for this initial validation study and its potential use in local or comparable service settings. The application-related elements in the figure should be interpreted as potential planning uses rather than as evidence of implementation effectiveness or equity-related outcomes.

## 3 Results

### 3.1 Results of Delphi expert consultation

Fifteen experts completed both rounds of Delphi consultation. The panel included specialists from Ningxia, Tianjin, Henan, Zhejiang, and other provinces or municipalities across China. Their professional fields covered general practice, pediatrics, obstetrics and gynecology, geriatrics, hospice and palliative care, nursing management, psychology, public health, and health education and science communication, thereby corresponding to the main age-specific, context-specific, psychosocial, and communication-preference modules of the scale. Pediatric and child-health expertise supported review of the infancy and toddlerhood, preschool-age, school-age, and adolescence modules; obstetrics and gynecology expertise supported review of the pregnancy and maternity module; geriatrics, general practice, nursing, and hospice and palliative care expertise supported review of the older adulthood and hospice and palliative care modules; and public health, psychology, and health education and science communication expertise supported review of the overall conceptual framework, psychological and social dimensions, and the two preference modules. The expert panel consisted of four men and 11 women, with a mean age of 46.8 ± 6.4 years and a mean of 19.5 ± 7.2 years of clinical or academic experience. Regarding educational attainment, four experts held a bachelor’s degree, six held a master’s degree, and five held a doctoral degree. Six experts had senior professional titles, five held associate senior professional titles, and four held intermediate professional titles. In both rounds, 15 questionnaires were distributed and all were returned, yielding a response rate of 100%, which indicated a high level of expert engagement. The expert authority coefficients (Cr) were 0.86 and 0.88 in the first and second rounds, respectively. Kendall’s coefficients of concordance (*W*) were 0.36 and 0.39, respectively, and both were statistically significant (*p* < 0.001), indicating acceptable expert authority and statistically significant convergence of expert opinion.

The initial Delphi item pool contained 94 items across 10 modules. Following the first round, the mean item importance scores ranged from 3.80 to 4.90, with coefficients of variation ranging from 0.06 to 0.21. Based on the experts’ written feedback, the following revisions were made: (1) module names were standardized, including revising “working period” to “adulthood,” replacing the colloquial term “silver age” with “older adulthood,” and revising “hospice period” to “hospice and palliative care”; (2) target respondents and response versions were clarified for each module; (3) item wording was revised to focus on health education and science communication needs rather than clinical diagnosis, treatment procedures, or objective knowledge testing; and (4) overlapping or overly narrow items were identified for merging or deletion to reduce respondent burden while preserving conceptual coverage.

Following the second round, the mean item importance scores ranged from 4.20 to 5.00, with coefficients of variation ranging from 0.06 to 0.17, suggesting further convergence of expert opinion. The remaining comments primarily concerned wording refinement, dimensional attribution, and reduction of overlap. The transparent item revision trail from 94 to 90 items was as follows: the infancy and toddlerhood, preschool-age, school-age, adolescence, format preference, and channel preference modules retained their original number of items; the adulthood module was reduced from 11 to 10 items by merging overlapping social-dimension items on workplace interpersonal relationships, social network expansion, community participation, family-role adaptation, intimate relationships, and health information appraisal; the pregnancy and maternity module was reduced from 13 to 12 items by deleting the item on maternal and infant product safety, which experts considered less central to health education and science communication needs than prenatal care, postpartum recovery, newborn care, psychological adaptation, family support, and workplace rights; the older adulthood module was reduced from 11 to 10 items by streamlining overlapping content on social participation, long-term care planning, family support, and later-life preparation; and the hospice and palliative care module was reduced from 9 to 8 items by merging family caregiver support, grief counseling, and linkage to hospice, legal, and social resources into a single social-dimension item. After these revisions, the final scale comprised 10 modules and 90 items: six age-specific modules, two context-specific modules, and two preference modules. The age-specific and context-specific modules were each structured around three theoretically defined dimensions: physiological, psychological, and social needs. The final item list is provided in Supplementary Table 1.

### 3.2 Sample flow and general characteristics of participants

The formal survey was conducted in two independent stages, and the detailed sample flow is presented in Supplementary Table 5. In Stage 1, 568 individuals were approached, of whom 545 completed eligibility screening and 520 provided informed consent and were enrolled. After data-quality screening, 33 questionnaires were excluded, including 8 because of missing key variables or excessive item-level missingness, 5 because of unresolvable outliers or logical inconsistencies, and 20 because of module-selection errors. Finally, 487 valid responses were included in item analysis and exploratory factor analysis.

In Stage 2, 385 individuals were approached, of whom 372 completed eligibility screening and 350 provided informed consent and were enrolled. After data-quality screening, 22 questionnaires were excluded, including 6 because of missing key variables or excessive item-level missingness, 3 because of unresolvable outliers or logical inconsistencies, and 13 because of module-selection errors. Finally, 328 valid responses were included in confirmatory factor analysis. Across the two stages, 953 individuals were approached, 917 were screened, 870 were enrolled and received questionnaires, and 815 were included in the final analytic sample, yielding a valid response rate of 93.7% among distributed questionnaires.

Among the final analytic sample, 312 respondents were men (38.3%) and 503 were women (61.7%). The respondents ranged in age from 18 to 85 years, with a mean age of 44.6 ± 15.2 years. Regarding educational attainment, 338 respondents (41.5%) had tertiary education or above, whereas 477 respondents (58.5%) had below-tertiary education. The sample distribution across modules in the two-stage survey is presented in Table 2.

**Table 2:** Module-specific sample distribution in the exploratory and confirmatory validation stages.

Module category	Module	EFA-stage sample size (*n* = 487)	CFA-stage sample size (*n* = 328)	Total across two stages (*n*)
Age-specific module	Infancy and toddlerhood (<3 years)	75	50	125
Age-specific module	Preschool age (3 to <7 years)	60	40	100
Age-specific module	School age (7 to <10 years)	70	45	115
Age-specific module	Adolescence (10 to <18 years)	55	37	92
Age-specific module	Adulthood (18 to <60 years)	120	80	200
Age-specific module	Older adulthood (≥60 years)	107	76	183
Age-specific module	Total age-specific modules	487	328	815
Context-specific module	Pregnancy and maternity	65	45	110
Context-specific module	Hospice and palliative care	40	28	68
Preference module	Format preference	487	328	815
Preference module	Channel preference	487	328	815

EFA, exploratory factor analysis; CFA, confirmatory factor analysis; n, number of valid respondents. Each respondent completed one age-specific module only; therefore, the total number of age-specific module responses was equal to the total analytic sample size. Context-specific modules were completed only by eligible respondents, whereas the Format Preference and Channel Preference modules were completed by all respondents. The CFA-stage n values were verified against the module-specific denominators used in Table 5.

The EFA-stage and CFA-stage samples were broadly comparable across predefined demographic, contextual, and module-membership characteristics (Supplementary Table 6). No significant between-stage differences were observed in respondent age, sex, educational attainment, self-rated health status, survey setting, age-specific module membership, or completion of the pregnancy/maternity or hospice and palliative care modules (all *p* > 0.05). Both preference modules were completed by all respondents and were therefore not statistically compared.

Module-specific demographic and contextual characteristics, including respondent type and administration mode, are shown in Supplementary Table 4. The six age-specific modules were mutually exclusive, whereas the pregnancy and maternity module and the hospice and palliative care module were context-specific modules completed only by eligible respondents. The two preference modules were completed by all respondents in each stage. For proxy or hybrid modules, respondent characteristics were reported for the actual respondent or caregiver unless otherwise specified.

### 3.3 Item analysis

The item-total correlation analysis showed that all items were positively correlated with the total score of their corresponding module, with correlation coefficients ranging from 0.52 to 0.89 (all *p* < 0.05). The critical ratio analysis showed statistically significant differences between the high-score and low-score groups for all items, with critical ratio values ranging from 3.12 to 8.45 (all *p* < 0.01), indicating satisfactory item discrimination. The analysis of Cronbach’s alpha coefficients after item deletion showed that deleting any item did not substantially increase the Cronbach’s alpha coefficient of any module, suggesting adequate internal consistency between each item and its corresponding module. Therefore, all 90 items were retained in the final version of the proposed scale.

### 3.4 Validity analysis

#### 3.4.1 Content validity

Content validity was evaluated based on the item-importance ratings from the two rounds of Delphi expert consultation. The 15 experts rated the importance of each item using a 5-point Likert scale, and ratings of 4 or 5 were treated as evidence that the item was important and relevant to the construct of perceived health education and science communication needs. Wording clarity, target-respondent appropriateness, content comprehensiveness, duplicate content, and missing content were assessed through written expert comments and incorporated into item revision. The I-CVI ranged from 0.87 to 1.00, indicating that 13 to 15 of the 15 experts rated each retained item as important. The S-CVI/Ave was 0.96. The modified kappa values ranged from 0.87 to 1.00 after correction for chance agreement, further supporting satisfactory content validity.

#### 3.4.2 Construct validity

##### 3.4.2.1 Exploratory factor analysis

Using the infancy and toddlerhood module as an illustrative module-level example (Table 3), principal axis factoring with Promax oblique rotation extracted three correlated factors corresponding to physiological, psychological, and social needs. This factor-retention decision was supported by a combined framework including eigenvalues >1, parallel analysis, scree plot inspection, theoretical interpretability, cross-loading diagnostics, and residual correlation diagnostics. The cumulative variance explained was 63.86%, communalities ranged from 0.410 to 0.760, and primary pattern coefficients ranged from 0.601 to 0.868. No item showed problematic cross-loading, and residual diagnostics did not indicate large or systematically clustered residual correlations suggesting the need for an additional factor. The inter-factor correlations supported the appropriateness of oblique rotation (see Table 4).

**Table 3:** Item-level exploratory factor analysis results for the infancy and toddlerhood module.

Item code	Item content	Physiological factor	Psychological factor	Social factor	Communality
Y1	Evidence-based feeding knowledge for infants and toddlers (breastfeeding, formula feeding, and complementary feeding methods)	**0.724**	0.118	−0.044	0.563
Y2	Growth and developmental monitoring and immunization guidance	**0.601**	0.074	0.136	0.410
Y3	Prevention of common diseases and home-based care for infants and toddlers	**0.798**	−0.062	0.091	0.673
Y4	Home safety protection and basic first aid	**0.748**	0.142	0.057	0.576
Y5	Sleep safety and establishment of daily routines	0.083	**0.806**	−0.105	0.718
Y6	Early emotion recognition and responsive caregiving	0.159	**0.696**	0.046	0.695
Y7	Sensory-cognitive stimulation and age-appropriate play guidance	−0.037	**0.868**	0.124	0.760
Y8	Promotion of language development and parent–child communication	0.096	0.173	**0.820**	0.687
Y9	Development of parent–child attachment and high-quality interaction	0.128	−0.069	**0.722**	0.588
Y10	Family support systems and access to parenting resources	0.051	0.149	**0.836**	0.715

Factor correlation matrix for the infancy and toddlerhood module			
Dimension	Physiological	Psychological	Social
Physiological	**1.000**		
Psychological	0.38	**1.000**	
Social	0.32	0.42	**1.000**

Principal axis factoring with Promax oblique rotation was used. Values are pattern coefficients. The three factors correspond to physiological, psychological, and social needs. Complete item-level exploratory factor analysis results for all modules are provided in Supplementary Table 9.

Values are inter-factor correlations from the Promax-rotated exploratory factor analysis solution. The diagonal values are fixed at 1.000. These exploratory factor correlations are distinct from the CFA latent-factor correlations used for discriminant validity assessment.

Bold values indicate the highest absolute pattern coefficient for each item and identify its primary factor loading.

**Table 4:** Module-specific exploratory factor analysis results using principal axis factoring.

Module	*n*	KMO	Bartlett’s test of sphericity *χ*^2^(*df*)	No. of extracted factors	Cumulative variance explained (%)	Range of communalities	Range of primary/factor coefficients	Range of factor correlations (*φ*)
Infancy and toddlerhood	75	0.708	206.740(45)***	3	63.86	0.410–0.760	0.601–0.868	0.32–0.42
Preschool age	60	0.770	216.254(45)***	3	69.00	0.489–0.794	0.672–0.862	0.34–0.48
School age	70	0.770	325.060(45)***	3	74.23	0.662–0.860	0.751–0.879	0.38–0.52
Adolescence	55	0.783	261.266(45)***	3	75.44	0.615–0.835	0.742–0.883	0.40–0.55
Adulthood	120	0.792	433.370(45)***	3	70.13	0.638–0.742	0.787–0.852	0.36–0.50
Older adulthood	107	0.771	489.040(45)***	3	73.54	0.646–0.818	0.771–0.900	0.38–0.52
Pregnancy and maternity	65	0.797	359.384(66)***	3	69.75	0.591–0.816	0.731–0.878	0.35–0.52
Hospice and palliative care	40	0.706	128.540(28)***	3	68.92	0.417–0.668	0.630–0.871	0.30–0.48
Format preference	487	0.864	867.049(10)***	1	61.31	0.599–0.630	0.774–0.794	—
Channel preference	487	0.851	803.752(10)***	1	59.47	0.535–0.652	0.732–0.808	—

EFA, exploratory factor analysis; KMO, Kaiser–Meyer–Olkin measure of sampling adequacy; *χ*^2^, chi-square statistic; df, degrees of freedom; φ, factor correlation. Principal axis factoring was used for factor extraction. Promax oblique rotation was applied to age-specific and context-specific modules, whereas no rotation was applied to the single-factor preference modules. Factor retention was based on parallel analysis, scree plot inspection, eigenvalues >1, theoretical interpretability, cross-loading diagnostics, and residual diagnostics. Bartlett’s test degrees of freedom were calculated as *p*(*p* − 1)/2, where p denotes the number of items in the module: df = 45 for 10-item modules, df = 66 for the 12-item Pregnancy and Maternity module, df = 28 for the 8-item Hospice and Palliative Care module, and df = 10 for the 5-item preference modules. ***p < 0.001.

Across the age-specific modules and the pregnancy and maternity module, the factor-retention framework supported three theoretically interpretable factors corresponding to physiological, psychological, and social needs. In the hospice and palliative care module, the EFA solution was also consistent with the predefined physiological, psychological, and social structure; however, the social domain was represented by a single item. Therefore, this item was retained to preserve theoretically important social-support content but was treated descriptively in subsequent CFA and subdimension-level reliability analyses. The cumulative variance explained ranged from 63.86 to 75.44%, communalities ranged from 0.410 to 0.860, and primary pattern coefficients ranged from 0.601 to 0.900. Cross-loading diagnostics showed no item requiring reassignment or deletion. For the two preference modules, parallel analysis, scree plot inspection, and theoretical interpretability supported a single-factor solution for each module. The format and channel preference modules explained 61.31 and 59.47% of the variance, respectively; across these two modules, factor coefficients ranged from 0.732 to 0.808 and communalities ranged from 0.535 to 0.652. These results suggested that the preference items within each module formed a coherent item set. However, because the purpose of these modules was to guide practical communication planning, the results should not be interpreted as requiring reliance on total scores. Item-level scores and rank-ordered preference profiles remain the primary basis for identifying preferred communication formats and channels. The detailed item-level EFA results are presented in Supplementary Table 9.

##### 3.4.2.2 Confirmatory factor analysis

The model fit indices for each module are shown in Table 5. To avoid interpreting global fit indices in isolation, CFA results were evaluated together with item-level standardized factor loadings, standard errors, squared multiple correlations, latent factor correlations, residual correlations, and the presence or absence of *post hoc* model modifications. Item-level standardized factor loadings, standard errors, z values, *p* values, and squared multiple correlations are presented in Supplementary Table 2. Across the estimated latent indicators, standardized factor loadings ranged from 0.64 to 0.88, with standard errors ranging from 0.04 to 0.09, and all estimated loadings were statistically significant. In the hospice and palliative care module, A8 was not interpreted as an indicator of a fully estimated latent social factor because it was the only item in that domain. Accordingly, no standardized loading, residual variance, reliability-based constraint, AVE, CR, or latent-factor correlation was interpreted for a social latent factor in this module. The item was reported as a descriptive observed indicator reflecting social and resource-linkage needs. No *post hoc* correlated error terms were added. Except for the prespecified descriptive treatment of the single social item in the hospice and palliative care module, the CFA models followed the predefined theoretical structure.

**Table 5:** Module-specific confirmatory factor analysis fit indices.

Module	*n*	*χ*^2^/*df*	RMSEA	SRMR	CFI	TLI	IFI
Infancy and toddlerhood	50	1.85	0.051	0.042	0.972	0.961	0.970
Preschool age	40	1.62	0.044	0.038	0.983	0.970	0.982
School age	45	1.78	0.049	0.041	0.971	0.962	0.973
Adolescence	37	2.12	0.058	0.048	0.962	0.953	0.964
Adulthood	80	1.95	0.054	0.045	0.973	0.965	0.973
Older adulthood	76	2.03	0.056	0.046	0.962	0.950	0.963
Pregnancy and maternity	45	2.41	0.068	0.055	0.951	0.942	0.954
Hospice and palliative care^†^	28	1.72	0.047	0.039	0.983	0.973	0.981
Format preference	328	1.28	0.031	0.028	0.994	0.984	0.995
Channel preference	328	1.35	0.035	0.030	0.983	0.974	0.983

CFA, confirmatory factor analysis; n, module-specific CFA-stage sample size; *χ*^2^/df, chi-square to degrees-of-freedom ratio; RMSEA, root mean square error of approximation; SRMR, standardized root mean square residual; CFI, comparative fit index; TLI, Tucker–Lewis index; IFI, incremental fit index. The *n* values correspond to the Stage 2 respondents who completed each module and were verified against Table 2. All CFA models were estimated using normal-theory maximum likelihood. Because several modules had small CFA-stage samples and showed evidence of multivariate non-normality, chi-square statistics, standard errors, and model-fit indices should be interpreted as approximate and preliminary internal-structure evidence rather than definitive confirmation of model fit. †In the hospice and palliative care module, the retained social domain consisted of a single item concerning family caregiving support, grief counseling, and linkage to resources. Therefore, this domain should be interpreted as a theoretically retained descriptive domain rather than as a fully evaluated multi-item factor or as a validated single-indicator latent factor. The reported global fit indices should be interpreted as preliminary internal-structure evidence for the module, mainly reflecting the evaluated physiological and psychological domains together with the overall module structure. This single social item was retained for descriptive item-level interpretation and module-level scoring, but AVE, CR, discriminant validity, and subdimension-level reliability were not calculated for this domain.

Although the global fit indices were within commonly accepted ranges, they should be interpreted cautiously because all CFA models were estimated using ML under five-category Likert-type responses. Distributional diagnostics showed no severe univariate non-normality, but Mardia’s multivariate kurtosis critical ratios indicated multivariate non-normality across modules. Because robust standard errors, scaled chi-square statistics, ordinal estimators, and estimator-based sensitivity analyses were not used, the reported standard errors, chi-square statistics, and model-fit indices may be affected by non-normality. This concern is particularly relevant for the smaller module-specific CFA samples. Accordingly, the *χ*^2^/df, RMSEA, SRMR, CFI, TLI, and IFI values should be interpreted as preliminary internal-structure evidence rather than as definitive confirmation of construct validity. Therefore, the CFA findings were interpreted as preliminary evidence supporting the plausibility of the proposed module structures, not as definitive confirmation of construct validity.

Convergent validity was further assessed using the average variance extracted (AVE) and composite reliability (CR), which were calculated from the standardized factor loadings in Supplementary Table 2 using the Fornell-Larcker formulas. Discriminant validity was assessed using the Fornell–Larcker criterion. Specifically, the square root of the AVE for each factor was compared with the CFA latent-factor correlations. These CFA latent-factor correlations were distinct from the EFA factor correlations obtained from Promax rotation. The discriminant validity matrix for the infancy and toddlerhood module is presented in Table 6, and the convergent and discriminant validity results across modules are shown in Table 7.

**Table 6:** Fornell–Larcker discriminant validity matrix for the infancy and toddlerhood module.

Dimension	Physiological	Psychological	Social
Physiological	**0.73**		
Psychological	0.45	**0.85**	
Social	0.38	0.42	**0.82**

Bold diagonal values are the square roots of the average variance extracted. Off-diagonal values are CFA latent-factor correlations. Discriminant validity was considered supported when the square root of AVE for each factor exceeded its correlations with other factors.

**Table 7:** Module-specific convergent and discriminant validity results.

Module	Physiological AVE	Physiological *CR*	Psychological AVE	Psychological *CR*	Social AVE	Social *CR*	Maximum inter-factor *r*	Minimum square root of AVE
Infancy and toddlerhood	0.54	0.82	0.72	0.89	0.67	0.86	0.45	0.73
Preschool age	0.58	0.85	0.70	0.88	0.62	0.83	0.42	0.76
School age	0.62	0.87	0.74	0.90	0.66	0.85	0.48	0.79
Adolescence	0.60	0.86	0.72	0.89	0.64	0.84	0.50	0.77
Adulthood	0.61	0.89	0.72	0.89	0.63	0.78	0.46	0.78
Older adulthood	0.63	0.90	0.71	0.88	0.65	0.79	0.44	0.79
Pregnancy and maternity	0.61	0.92	0.72	0.89	0.63	0.78	0.52	0.78
Hospice and palliative care	0.55	0.86	0.69	0.82	—	—	0.40	0.74
Format preference	0.63	0.89	—	—	—	—	—	—
Channel preference	0.61	0.89	—	—	—	—	—	—

AVE, average variance extracted; CR, composite reliability. Preference modules were modeled as single-factor preference-profile modules; therefore, discriminant validity was not applicable and is indicated by “—.” AVE and CR were calculated from standardized CFA factor loadings. For the hospice and palliative care module, the retained social domain consisted of a single item and was interpreted as a theoretically retained descriptive domain rather than as a fully evaluated multi-item factor. Therefore, AVE, CR, discriminant validity, and subdimension-level reliability were not calculated for this domain. The absence of these estimates should not be interpreted as evidence against the theoretical relevance of social-support content; rather, it reflects the methodological limitation of evaluating a single-item domain within latent-variable validity analyses.

### 3.5 Reliability analysis

Reliability analysis was conducted using the combined formal survey samples from the EFA and CFA stages. Because the scale adopted a modular administration design, reliability coefficients were calculated separately for each module rather than for the full 90-item scale. At the module level, Cronbach’s alpha coefficients ranged from 0.80 to 0.87, with 95% confidence intervals ranging from 0.76–0.87 to 0.83–0.90. McDonald’s omega coefficients ranged from 0.81 to 0.88, Spearman–Brown corrected split-half reliability coefficients ranged from 0.77 to 0.84, and corrected item–total correlations ranged from 0.52 to 0.82. These findings indicated acceptable internal consistency reliability for all modules (Table 8).

**Table 8:** Module-level internal consistency reliability results.

Module	*n*	No. of items	Cronbach’s alpha	95% CI for Cronbach’s alpha	McDonald’s omega	Spearman–Brown split-half reliability	Range of corrected item-total correlations
Infancy and toddlerhood	125	10	0.85	0.81–0.89	0.86	0.82	0.52–0.78
Preschool age	100	10	0.83	0.78–0.87	0.84	0.80	0.54–0.76
School age	115	10	0.84	0.80–0.88	0.85	0.81	0.55–0.79
Adolescence	92	10	0.82	0.76–0.87	0.83	0.79	0.52–0.77
Adulthood	200	10	0.86	0.83–0.89	0.87	0.83	0.58–0.82
Older adulthood	183	10	0.85	0.82–0.88	0.86	0.82	0.56–0.80
Pregnancy and maternity	110	12	0.87	0.83–0.90	0.88	0.84	0.55–0.81
Hospice and palliative care	68	8	0.84	0.78–0.89	0.85	0.81	0.54–0.79
Format preference	815	5	0.81	0.79–0.83	0.82	0.78	0.56–0.72
Channel preference	815	5	0.80	0.78–0.82	0.81	0.77	0.54–0.70

*n*, number of valid respondents who completed each module in the combined formal sample across the EFA and CFA stages; CI, confidence interval; α, Cronbach’s alpha; ω, McDonald’s omega. The 95% CI refers to Cronbach’s alpha. Corrected item–total correlations were calculated between each item and its corresponding module total score after excluding that item. Because the scale used a modular administration design, total-scale reliability across all 90 items was not calculated.

Subdimension-level reliability was further examined for the physiological, psychological, and social subdimensions of the age-specific and context-specific modules where each subdimension contained at least two items. Across the multi-item subdimensions, Cronbach’s alpha coefficients ranged from 0.78 to 0.84, McDonald’s omega coefficients ranged from 0.78 to 0.85, and corrected item–subdimension-total correlations ranged from 0.60 to 0.82, indicating acceptable internal consistency. For two-item subdimensions, reliability estimates were interpreted cautiously, and the corrected item–subdimension-total correlation was reported as the single inter-item correlation. The single-item social domain of the hospice and palliative care module was not included in subdimension-level reliability estimation. No assumed reliability coefficient was assigned to this item, and no reliability-based residual variance constraint was used to identify a single-indicator latent factor. The item was therefore interpreted descriptively and retained for item-level interpretation and module-level scoring. Detailed results are presented in Supplementary Table 11.

### 3.6 Final scale

The final Modular Life-Course Health Education and Science Communication Needs Scale comprised 10 modules and 90 items. For the age-specific and context-specific modules, higher scores indicated greater perceived needs for the corresponding health education and science communication content. These scores were intended to reflect respondents’ self-reported or caregiver-reported priorities for educational content and should not be interpreted as objectively assessed knowledge deficits. Because perceived-need items may elicit broad endorsement of socially valued health topics, high scores should be interpreted together with item-level distributions, ceiling effects, and rank-ordered item profiles, rather than as definitive evidence that all highly rated topics have the same intervention priority. For the preference modules, higher item scores indicated stronger preference or receptivity toward the corresponding health science communication format or channel. In practical interpretation, item-level profiles and priority rankings should be used to identify the most acceptable communication options for a given population or service context. Module mean scores may be reported as supplementary indicators of overall receptivity but should not replace item-level interpretation. The complete final scale and administration guide are provided in Supplementary Table 1, including all final items, response options, module-selection rules, scoring procedures, interpretation guidance, and instructions for self-report, caregiver-report, and hybrid administration. Scores obtained from different response or administration modes should be interpreted in light of the respondent context, because measurement equivalence across self-report, caregiver proxy report, hybrid child–caregiver completion, and investigator-administered completion was not directly tested in this initial validation study.

## 4 Discussion

### 4.1 Innovation and practical value of the modular design

The modular design of the proposed scale may provide a structured framework for life-course-oriented needs assessment. Compared with instruments focused mainly on older adults or reproductive-age populations (30, 31), this scale covers six age-specific modules and two context-specific modules. The adulthood module was designed as a broad module for individuals aged 18 to <60 years to capture cross-cutting adult needs, including chronic disease prevention, occupational health, sleep health, work–life balance, family role adaptation, social support, and health information appraisal (32, 33). However, this design should not be interpreted as evidence of homogeneity across adulthood. Early adulthood and middle adulthood may differ in developmental tasks, occupational trajectories, caregiving roles, and health priorities; therefore, further refinement into adult submodules may be warranted.

The integration of age-specific, context-specific, and preference modules allows respondents to complete items relevant to their life stage, health context, and communication preferences, thereby reducing unnecessary response burden. The context-specific modules capture needs associated with pregnancy, maternity, hospice care, and palliative care, whereas the preference modules identify preferred formats and channels for receiving health information. Because communication formats and channels are often complementary rather than mutually exclusive, these modules should be interpreted mainly through item-level profiles and priority rankings rather than total scores alone. Overall, the modular structure offers flexibility for local needs assessment and future adaptation, but it should be regarded as preliminary input for health education planning rather than evidence that scale-guided services improve health literacy outcomes.

### 4.2 Evaluation of reliability and validity

Overall, the psychometric results provided preliminary evidence for the internal structure, content validity, convergent and discriminant validity, and internal consistency reliability of the proposed scale. These findings should be interpreted in relation to the construct actually assessed: perceived health education and science communication needs and communication preferences. The study did not examine objectively measured knowledge deficits, clinical risk profiles, or externally assessed educational needs; nor did it assess test–retest reliability or criterion-related validity. EFA using principal axis factoring with Promax rotation supported correlated physiological, psychological, and social factors across the age-specific and context-specific modules, consistent with the theoretical assumption that these needs are interrelated. Factor-retention decisions were further supported by parallel analysis, scree plot inspection, cross-loading diagnostics, and residual correlation diagnostics. CFA in an independent validation sample provided preliminary evidence that the hypothesized module structures were empirically plausible, but the results should not be interpreted as definitive confirmation because several module-specific CFA samples were small. Content validity was satisfactory, with an S-CVI/Ave of 0.96, and internal consistency reliability was acceptable to good, with Cronbach’s alpha coefficients ranging from 0.80 to 0.87.

The preference modules should also be interpreted according to their intended pragmatic use. Although their Cronbach’s alpha coefficients ranged from 0.80 to 0.81, communication preferences are influenced by digital access, media habits, trust in information sources, service availability, and contextual constraints. Therefore, these modules are most useful for identifying prioritized formats and channels at the item level rather than for interpreting a total score alone.

The CFA findings should be interpreted cautiously because the modular design required each CFA model to be estimated using only the subgroup that completed the corresponding module. Several CFA subsamples were small, including preschool age, school age, adolescence, pregnancy and maternity, and especially hospice and palliative care. Consequently, standardized loadings, standard errors, factor correlations, AVE, CR, and global fit indices may be unstable. The hospice and palliative care module should therefore be regarded as providing exploratory internal-structure evidence only, and larger targeted samples are needed to confirm its factor structure and score interpretation.

Finally, test–retest reliability and criterion-related validity were not evaluated. Internal consistency estimates do not demonstrate that scores remain stable over time, and no external validated measures of health literacy, digital health literacy, health education needs, health information appraisal, patient activation, or behavioral outcomes were administered. Therefore, future studies should examine temporal stability, concurrent validity, and predictive validity before the scale is used for longitudinal monitoring, intervention evaluation, or external outcome prediction.

### 4.3 Potential implications for context-sensitive health literacy planning

Within the context of this single-municipality initial validation study, the proposed scale may provide structured respondent-informed information for context-sensitive health literacy planning. By allowing individuals and caregivers at different life stages to report perceived content needs and communication preferences, the scale may help local services identify mismatches between population needs and available health education resources. It may also support more tailored planning in healthcare institutions, schools, workplaces, communities, long-term care settings, and hospice and palliative care services with demographic and service characteristics similar to those of the present sample. However, these implications remain preliminary and geographically bounded. The study did not directly evaluate community participation, shared decision-making, co-design, empowerment outcomes, resource allocation, disparity reduction, or improved access for underserved groups.

The flexible administration design may broaden participation in needs assessment, but it should not be interpreted as direct evidence of inclusion. Self-report, caregiver-report, and joint child–caregiver assisted formats allowed data collection from groups that are often difficult to include in conventional questionnaire studies. However, the infancy/toddlerhood and preschool-age modules relied on caregiver proxy reporting, and the respondent-informed nature of these modules should therefore be understood as caregiver-mediated rather than fully child-reported. Future studies should examine proxy-response effects and develop age-appropriate approaches to better capture children’s own perspectives.

Overall, the scale should be viewed primarily as a preliminary psychometric instrument for structured needs assessment. It may help identify priority topics, compare perceived needs across modules or dimensions, and align educational content with preferred communication formats and channels. However, the present study did not test whether scale-guided interventions improve health literacy, health information appraisal, service use, behavioral outcomes, or equity-related outcomes. Future implementation studies should evaluate these applications using longitudinal or controlled designs.

### 4.4 Limitations and future directions

This study has several limitations. First, all participants were recruited through convenience sampling from Tianjin-based medical, community, eldercare, and hospice/palliative care settings. Although respondents represented different life stages and health contexts, the sample cannot represent the diversity of geographic regions, rural–urban environments, socioeconomic conditions, cultural backgrounds, institutional resources, and health communication ecosystems in China or internationally. Therefore, the findings should be interpreted as initial, geographically bounded psychometric evidence. Multicenter and cross-regional validation using more diverse and preferably stratified samples is required. Measurement invariance and differential item functioning should also be examined before the scale is used for subgroup comparison, equity monitoring, or broader cross-regional and cross-cultural assessment (34).

Second, the age-band boundaries, particularly the adulthood module, represent a structural limitation. The adulthood module covers individuals aged 18 to <60 years, a heterogeneous period involving different developmental tasks, occupational exposures, family responsibilities, caregiving roles, chronic disease risks, and communication preferences. Although this broad module was designed to capture cross-cutting adult needs and maintain administration feasibility, the present study did not establish that a single adulthood module is sufficient for all adult subgroups. Future studies should examine measurement invariance, differential item functioning, factor structure, reliability, and score interpretation across early- and middle-adulthood groups.

Third, several module-specific validation samples were small, particularly in the CFA stage. In addition, the CFA models were estimated using ML for five-category Likert-type items despite evidence of multivariate non-normality (37). Because robust standard errors, scaled chi-square statistics, ordinal-data estimators, and estimator-based sensitivity analyses were not used, model-fit indices, standard errors, chi-square statistics, standardized loadings, factor correlations, AVE, CR, and related estimates may be unstable or biased. This limitation is most pronounced for the hospice and palliative care module, with a CFA-stage subsample of 28. The social domain of this module was also represented by a single item and was therefore interpreted descriptively rather than as a fully validated latent domain. Larger targeted samples, expanded social-domain items, and sensitivity analyses using robust or ordinal estimators are needed in future validation studies.

Fourth, equivalence across respondent and administration modes was not examined. Responses may be influenced by self-report bias, recall bias, caregiver proxy interpretation, joint child–caregiver interaction, interviewer presence, ceiling effects, acquiescence, and social desirability (35). Because paired self-report and proxy-report data were not collected and administration modes were not randomized, equivalence among self-report, caregiver-report, hybrid, and investigator-administered responses could not be established. Future studies should examine administration-mode effects, item-level ceiling effects, and response-style diagnostics.

Fifth, test–retest reliability and criterion-related validity were not established. The cross-sectional design supports internal consistency evidence but not temporal stability. No external validated measure of health literacy, digital health literacy, health education needs, health information appraisal, patient activation, objective knowledge, or behavioral outcomes was administered; therefore, the study could not determine whether perceived-need scores correspond to external constructs, objective educational gaps, service use, or behavioral change. Future studies should examine test–retest reliability, concurrent validity, and predictive validity using appropriate external criteria and follow-up outcomes.

Sixth, the scale’s value for guiding intervention design and evaluation has not yet been empirically established. It can identify perceived needs and communication preferences, but this study did not assess whether scale-guided interventions improve health literacy, information appraisal, service engagement, behavioral outcomes, or equity-related outcomes. Future implementation studies should evaluate whether use of the scale in assessment, intervention tailoring, delivery, and reassessment improves the reach, acceptability, appropriateness, and effectiveness of health education and science communication activities.

## 5 Conclusion

The Modular Life-Course Health Education and Science Communication Needs Scale demonstrated acceptable initial psychometric performance in a Tianjin-based sample of Chinese clinical and community service users and primary caregivers. It provides preliminary evidence for content validity, internal structure, convergent and discriminant validity, and internal consistency reliability, and offers a modular framework for assessing perceived health education and science communication needs and communication preferences across life stages and caregiving contexts. However, the adulthood module spans 18 to <60 years, test–retest reliability and criterion-related validity were not evaluated, and findings for the hospice and palliative care module remain exploratory because of the small CFA subsample. These issues require further empirical evaluation.

Because all participants were recruited from a single municipality, the findings should be interpreted as preliminary and geographically bounded. The scale may serve as a candidate instrument for stage-specific and context-sensitive needs assessment in local or comparable Chinese service settings. Before broader implementation, further multicenter, cross-regional, rural–urban, cross-cultural, and measurement-equivalence validation is required. Direct empirical evidence is also needed before the scale can be described as supporting community empowerment, reducing inequities, improving inclusion, or evaluating intervention outcomes.

## Data Availability

The original contributions presented in the study are included in the article/Supplementary material, further inquiries can be directed to the corresponding author/s.
