# Supplementary material for: Development and initial validation of a modular life-course health education and science communication needs scale for Chinese clinical and community service users: a Tianjin-based study
Source: Front Public Health. 2026 Jul 23;14:1905218. doi: 10.3389/fpubh.2026.1905218 (PMC13443298; doi:10.3389/fpubh.2026.1905218)
Supplement: Supplementary file 1 [file Data_Sheet_1.PDF]

Supplementary Table 1. Final Modular Life-Course Health Education and Science Communication Needs Scale: Full Item List, Administration Instructions, and Scoring Guide

Purpose and scope

This scale is a modular needs-assessment instrument for evaluating perceived health education and science communication needs among Chinese clinical and community service users and their primary caregivers. In the present validation study, the empirical evidence was obtained from a Tianjin-based sample; therefore, the scale should be interpreted as an initially validated tool for local and similar service contexts rather than as a diagnostic instrument or a universally validated international measure.

Administration and scoring guide

Aspect	Specification
Construct assessed	The age-specific and context-specific modules assess respondents' perceived needs for health education and science communication. They do not directly assess objective clinical need, disease risk, health literacy level, intervention effectiveness, or readiness to change.
Item stem for content modules	Recommended stem: "To what extent do you, or the person under your care, need health education and science communication about the following topic?"
Item stem for preference modules	Recommended stem: "To what extent do you prefer the following format/channel when receiving health education and science communication?"
Module selection	Each respondent completes one age-specific module according to their own age or the age of the person under their care. Eligible respondents additionally complete the pregnancy and maternity module or the hospice and palliative care module. All respondents complete the format preference and channel preference modules.
Response options	A 5-point Likert response format is used. For content modules, response options are scored as 1 = no need at all, 2 = low need, 3 = moderate need, 4 = high need, and 5 = very high need. For preference modules, response options are scored as 1 = not preferred at all, 2 = low preference, 3 = moderate preference, 4 = high preference, and 5 = very high preference. Higher scores indicate greater perceived content need or stronger receptivity to the corresponding communication format or channel.
Scoring	For age-specific and context-specific modules, calculate the module mean score and dimension mean scores. Module total scores may also be reported when all items in that module are completed. For preference modules, calculate the module mean score and item-level scores to identify preferred formats or channels.
Interpretation	Scores should be interpreted within each module and respondent group. Because respondents complete different modules according to life stage or health context, an overall score across all 90 items should not be calculated.
Proxy and hybrid responses	Caregiver-report, self-report/caregiver-report, and hybrid versions are used when the target person cannot always complete a self-report independently. For proxy or hybrid modules, responses should be interpreted as the actual respondent's or caregiver-assisted assessment of perceived needs.

Module overview

Module type	Module code and name	Target respondents	Response version	No. items
Age-specific	Y. Infancy and toddlerhood	Caregivers of children aged <3 years	Caregiver-report	10
Age-specific	X. Preschool age	Caregivers of children aged 3 to <7 years	Caregiver-report	10
Age-specific	L. School age	Children aged 7 to <10 years and their caregivers	Hybrid	10
Age-specific	Q. Adolescence	Adolescents aged 10 to <18 years and their caregivers	Self-report/caregiver-report	10
Age-specific	Z. Adulthood	Adults aged 18 to <60 years	Self-report	10
Age-specific	S. Older adulthood	Older adults aged ≥60 years	Self-report	10
Context-specific	C. Pregnancy and maternity	Individuals from preconception to 1 year postpartum	Self-report/caregiver-report	12
Context-specific	A. Hospice and palliative care	Individuals receiving hospice and palliative care and their caregivers	Self-report/caregiver-report	8
Preference	F. Format preference	All respondents	As applicable	5
Preference	D. Channel preference	All respondents	As applicable	5

Full item list

Y. Infancy and toddlerhood module (10 items, caregiver-report version)
Target respondents: caregivers of children aged <3 years

Code	Dimension	Item content
Y1	Physiological	Evidence-based feeding for infants and toddlers (breastfeeding, formula feeding, and introduction of complementary foods)
Y2	Physiological	Growth and developmental monitoring and immunization guidance
Y3	Physiological	Prevention of common illnesses and home-based care for infants and toddlers
Y4	Physiological	Home safety protection and basic first aid
Y5	Psychological	Sleep safety and establishment of daily routines
Y6	Psychological	Early emotion recognition and responsive caregiving
Y7	Psychological	Sensory-cognitive stimulation and age-appropriate play guidance
Y8	Social	Promotion of language development and parent-child communication
Y9	Social	Development of parent-child attachment and high-quality interaction
Y10	Social	Family support systems and access to parenting resources

X. Preschool-age module (10 items, caregiver-report version)
Target respondents: caregivers of children aged 3 to <7 years

Code	Dimension	Item content
X1	Physiological	Guidance on motor development (gross motor activities, fine motor training, and physical play)
X2	Physiological	Prevention of infectious diseases and adaptation to kindergarten (hand, foot, and mouth disease; influenza; varicella prevention and control; and school-entry preparation)
X3	Physiological	Balanced nutrition and development of healthy eating habits (dietary planning, correction of picky eating, snack management, and food safety)
X4	Physiological	Oral health and vision protection (dental caries prevention, eye hygiene, and management of electronic device use)
X5	Psychological	Self-awareness and emotion management skills (self-concept, emotional expression, and coping with frustration)
X6	Psychological	Early development of learning dispositions (attention development, preservation of curiosity, and task persistence)
X7	Psychological	Adaptation to kindergarten or school entry and management of separation anxiety
X8	Social	Peer interaction and social skills (sharing, cooperation, conflict resolution, and friendship formation)
X9	Social	Outdoor activity safety protection (traffic safety, prevention of child abduction, and prevention of sports injuries)
X10	Social	Early development of family rules and social norms (behavioral habits, public etiquette, and rule awareness)

L. School-age module (10 items, hybrid version)
Target respondents: children aged 7 to <10 years and their caregivers

Code	Dimension	Item content
L1	Physiological	Vision protection and spinal health (myopia prevention and control, proper sitting posture, and scoliosis screening)
L2	Physiological	Balanced nutrition and weight management (nutritious breakfast, snack choices, obesity prevention, and growth and developmental monitoring)
L3	Physiological	Maintenance of oral health (dental caries prevention, timing of orthodontic treatment, and oral hygiene habits)
L4	Physiological	Physical fitness enhancement and motor skills (fitness training, selection of sports activities, and prevention of sports injuries)
L5	Psychological	Development of study habits and learning motivation (stress recognition, time management, and learning strategies)
L6	Psychological	Self-identity and confidence building (strengths identification, self-efficacy, and resilience)
L7	Psychological	Emotion regulation and mental health (recognition of anxiety and depression, emotion management, and pathways for psychological support)
L8	Social	Peer relationships and responses to school bullying (friendship maintenance, interpersonal conflict, and bullying identification and response)
L9	Social	Internet literacy and digital health (management of electronic device use, online safety, and information appraisal)
L10	Social	Family communication and quality of parent-child interaction (effective communication, boundary setting, and shared activities)

Q. Adolescence module (10 items, self-report/caregiver-report version)
Target respondents: adolescents aged 10 to <18 years and their caregivers

Code	Dimension	Item content
Q1	Physiological	Puberty sexuality education and reproductive health (development of secondary sexual characteristics, menstruation, nocturnal emissions, sexual health knowledge, and prevention of sexually transmitted infections)
Q2	Physiological	Posture management and protection from sports injuries (postural correction, evidence-based exercise, injury prevention, and first aid)
Q3	Physiological	Sleep health management (interventions for insufficient sleep, regular daily schedules, and improvement of sleep quality)
Q4	Physiological	Nutritional needs and weight management (adolescent nutrition, weight control, and prevention of eating disorders)
Q5	Psychological	Mental health and stress coping (emotion regulation, stress management, and identification of psychological crises)
Q6	Psychological	Prevention of internet addiction and promotion of digital health (gaming addiction, social media dependence, and digital literacy)
Q7	Psychological	Self-identity and career planning (self-exploration, vocational interests, and academic planning)
Q8	Social	Parent-adolescent communication skills and family support (intergenerational communication, negotiation of autonomy, and family conflict resolution)
Q9	Social	Peer influence and social skills (peer pressure, social decision-making, and healthy friendships)
Q10	Social	Identification of and responses to risk behaviors (substance use, self-harm behaviors, and responses to high-risk situations)

Z. Adulthood module (10 items, self-report version)
Target respondents: adults aged 18 to <60 years

Code	Dimension	Item content
Z1	Physiological	Prevention of common occupational health problems (cervical and lumbar spine disorders, repetitive strain injuries of the hand and wrist, sedentary behavior syndrome, and visual fatigue)
Z2	Physiological	Regular health checkups and early screening for chronic diseases (selection of checkup items, interpretation of examination reports, and cancer screening)
Z3	Physiological	Evidence-based exercise and weight management (exercise prescription, fat loss and muscle gain, weight control, and prevention of sports injuries)
Z4	Physiological	Healthy diet and nutritional supplementation (balanced diet, healthier food delivery choices, and appropriate use of dietary supplements)
Z5	Physiological	Sleep health management (sleep disorders, adjustment of daily routines, and improvement of sleep quality)
Z6	Psychological	Workplace stress management and psychological resilience (stress recognition, coping strategies, and development of psychological capital)
Z7	Psychological	Work-life balance and time management (boundary setting, energy management, and life satisfaction)
Z8	Psychological	Emotion regulation and maintenance of mental health (recognition of anxiety and depression, emotion management, and professional help-seeking)
Z9	Social	Interpersonal communication, social support, and community participation (collegial relationships, supervisor-subordinate communication, workplace conflict, social support networks, volunteering, and community resources)
Z10	Social	Adaptation to family roles, intimate relationships, and health information appraisal (romantic and marital relationships, parenting preparation, intergenerational relationships, identification of misinformation, and access to authoritative information)

S. Older adulthood module (10 items, self-report version)
Target respondents: older adults aged 60 years and older

Code	Dimension	Item content
S1	Physiological	Prevention and self-management of chronic diseases (long-term management of hypertension, diabetes, coronary heart disease, chronic obstructive pulmonary disease, and related conditions)
S2	Physiological	Fall prevention and rehabilitative exercise (fall risk assessment, home modification, balance training, and muscle strengthening)
S3	Physiological	Nutrition in older adulthood and appropriate medication use (dietary guidelines, prevention of malnutrition, polypharmacy management, drug interactions, and weight management)
S4	Physiological	Maintenance of cognitive function and dementia prevention (cognitive training, early identification, and intervention strategies)
S5	Physiological	Maintenance of sensory function (vision and hearing protection, oral health, and constipation management)
S6	Psychological	Emotion management and mental health (loneliness, depression and anxiety, active aging, and psychological adjustment)
S7	Psychological	Adjustment to retirement and reconstruction of personal value (retirement planning, role transition, social participation, and development of a sense of meaning)
S8	Psychological	End-of-life education and preparation (life review, advance directives, and preparation for a good death)
S9	Social	Social participation and community integration (community activities, volunteering, intergenerational interaction, and digital inclusion)
S10	Social	Long-term care and aging-related planning (care resources, selection of eldercare institutions, financial planning, and legal preparation)

C. Pregnancy and maternity module (12 items, self-report/caregiver-report version)
Target respondents: individuals from preconception to 1 year postpartum

Code	Dimension	Item content
C1	Physiological	Evidence-based pregnancy preparation and preconception care (preconception health assessment, folic acid supplementation, and lifestyle modification)
C2	Physiological	Nutrition and weight management during pregnancy (dietary guidelines, nutritional supplementation, weight control, and prevention of gestational diabetes)
C3	Physiological	Prenatal care and key points of antenatal visits (prenatal care procedures, recognition of abnormalities, and prevention of complications)
C4	Physiological	Childbirth preparation and labor management (choice of delivery mode, knowledge of labor analgesia, and cooperation during labor)
C5	Physiological	Postpartum rehabilitation and physical recovery (pelvic floor rehabilitation, body shape recovery, and puerperal care)
C6	Physiological	Newborn care and neonatal feeding (feeding techniques, common problems, and newborn care)
C7	Physiological	Emergency care knowledge during pregnancy and the postpartum period (recognition of postpartum hemorrhage, first aid for neonatal asphyxia, and management of precipitous labor)
C8	Psychological	Prenatal anxiety and emotion management (anxiety alleviation, emotion regulation, and psychological support)
C9	Psychological	Screening and intervention for postpartum depression (depression recognition, help-seeking pathways, and family support)
C10	Psychological	Role transition and psychological adaptation (maternal role identity, self-adjustment, and marital relationships)
C11	Social	Development of family support systems (partner involvement, coordination with grandparents, and social support resources)
C12	Social	Protection of workplace rights and return to work (maternity leave policies, breastfeeding rights, and career continuity)

A. Hospice and palliative care module (8 items, self-report/caregiver-report version)
Target respondents: individuals receiving hospice and palliative care and their caregivers

Code	Dimension	Item content
A1	Physiological	Pain assessment and effective relief (pain recognition, medication management, nonpharmacological therapies, and comfort care)
A2	Physiological	Skin and wound care (pressure injury prevention and stage-based management, incontinence-associated dermatitis, and wound management)
A3	Physiological	Nutritional support and management of eating difficulties (appetite enhancement, dysphagia, tube-feeding care, and hydration)
A4	Physiological	Comfortable positioning and daily care (positioning management, range-of-motion activities, oral care, and personal hygiene)
A5	Physiological	Dyspnea and symptom management (respiratory comfort, secretion management, and management of death rattle)
A6	Psychological	End-of-life psychological support and emotional counseling (death anxiety, life review, meaning-making, and spiritual care)
A7	Psychological	Respect for patient preferences and advance care planning (ACP) (preference communication, surrogate decision-making, and hospice and palliative care options)
A8	Social	Family caregiving support, grief counseling, and linkage to resources (caregiving skills training, respite services, anticipatory grief, bereavement support, hospice and palliative care resources, legal consultation, and funeral preparation)

F. Health education and science communication format preference module (5 items)
Target respondents: all respondents

Code	Item content
F1	Video-based health education and science communication (short videos, animated demonstrations, documentaries, and live streaming)
F2	Graphic and text-based health education and science communication (brochures, posters, WeChat Official Account articles, and infographics)
F3	Audio-based health education and science communication (podcasts, audiobooks, radio programs, and voice broadcasts)
F4	Offline activities (health lectures, free clinic consultations, workshops, and group activities)
F5	Personalized consultation (one-on-one consultation, health management apps, and intelligent question-answering)

D. Health education and science communication channel preference module (5 items)
Target respondents: all respondents

Code	Item content
D1	Digital media platforms (WeChat, Douyin/TikTok, Xiaohongshu/RED, Bilibili, and health-related apps)
D2	Community-based channels (neighborhood committees, community centers, senior activity centers, and Party-mass service centers)
D3	School- and workplace-based channels (school courses, workplace health lectures, and trade union activities)
D4	Interpersonal networks (family members and friends, colleagues, neighbors, and peer patient sharing)
D5	Healthcare institutions (hospitals, community health service centers, and clinic-based publicity)

Notes

- The final scale contains 10 modules and 90 items: six age-specific modules, two context-specific modules, and two communication preference modules.
- For age-specific and context-specific modules, the item content is organized into physiological, psychological, and social dimensions. The two preference modules are pragmatic preference-screening indices and are not intended to define mutually exclusive preference types.
- Context-specific modules may overlap with age-specific modules because they are completed only by eligible respondents. The two preference modules are completed by all respondents.
- The scale is intended to support needs assessment and tailored health education/science communication planning in clinical, community, school, workplace, eldercare, and hospice/palliative care service contexts. It should not be used as a stand-alone diagnostic, clinical triage, or intervention-effectiveness evaluation instrument without further validation.

Supplementary Table 2. Item-level standardized factor loadings, standard errors, significance values, and squared multiple correlations from confirmatory factor analysis across modules

Module	Item	Factor	Standardized loading (λ)	SE	z	*p*	R^2
Infancy and toddlerhood (n=50)	Y1	Physiological	0.71	0.08	8.88	<0.001	0.50
	Y2	Physiological	0.64	0.09	7.11	<0.001	0.41
	Y3	Physiological	0.82	0.06	13.67	<0.001	0.67
	Y4	Physiological	0.76	0.07	10.86	<0.001	0.58
	Y5	Psychological	0.85	0.05	17.00	<0.001	0.72
	Y6	Psychological	0.83	0.06	13.83	<0.001	0.69
	Y7	Psychological	0.87	0.05	17.40	<0.001	0.76
	Y8	Social	0.83	0.06	13.83	<0.001	0.69
	Y9	Social	0.77	0.07	11.00	<0.001	0.59
	Y10	Social	0.85	0.05	17.00	<0.001	0.72
Preschool age (n=40)	X1	Physiological	0.72	0.08	9.00	<0.001	0.52
	X2	Physiological	0.78	0.07	11.14	<0.001	0.61
	X3	Physiological	0.81	0.06	13.50	<0.001	0.66
	X4	Physiological	0.74	0.07	10.57	<0.001	0.55
	X5	Psychological	0.80	0.06	13.33	<0.001	0.64
	X6	Psychological	0.86	0.05	17.20	<0.001	0.74
	X7	Psychological	0.84	0.05	16.80	<0.001	0.71
	X8	Social	0.82	0.06	13.67	<0.001	0.67
	X9	Social	0.76	0.07	10.86	<0.001	0.58
	X10	Social	0.79	0.06	13.17	<0.001	0.62
School age (n=45)	L1	Physiological	0.75	0.07	10.71	<0.001	0.56
	L2	Physiological	0.80	0.06	13.33	<0.001	0.64
	L3	Physiological	0.77	0.07	11.00	<0.001	0.59

Module	Item	Factor	Standardized loading (λ)	SE	z	*p*	R^2
	L4	Physiological	0.82	0.06	13.67	<0.001	0.67
	L5	Psychological	0.84	0.05	16.80	<0.001	0.71
	L6	Psychological	0.86	0.05	17.20	<0.001	0.74
	L7	Psychological	0.88	0.04	22.00	<0.001	0.77
	L8	Social	0.81	0.06	13.50	<0.001	0.66
	L9	Social	0.79	0.06	13.17	<0.001	0.62
	L10	Social	0.83	0.05	16.60	<0.001	0.69
Adolescence (n=37)	Q1	Physiological	0.74	0.07	10.57	<0.001	0.55
	Q2	Physiological	0.79	0.06	13.17	<0.001	0.62
	Q3	Physiological	0.76	0.07	10.86	<0.001	0.58
	Q4	Physiological	0.81	0.06	13.50	<0.001	0.66
	Q5	Psychological	0.83	0.05	16.60	<0.001	0.69
	Q6	Psychological	0.85	0.05	17.00	<0.001	0.72
	Q7	Psychological	0.87	0.05	17.40	<0.001	0.76
	Q8	Social	0.80	0.06	13.33	<0.001	0.64
	Q9	Social	0.78	0.07	11.14	<0.001	0.61
	Q10	Social	0.82	0.06	13.67	<0.001	0.67
Adulthood (n=80)	Z1	Physiological	0.76	0.07	10.86	<0.001	0.58
	Z2	Physiological	0.80	0.06	13.33	<0.001	0.64
	Z3	Physiological	0.82	0.06	13.67	<0.001	0.67
	Z4	Physiological	0.78	0.07	11.14	<0.001	0.61
	Z5	Physiological	0.75	0.07	10.71	<0.001	0.56
	Z6	Psychological	0.84	0.05	16.80	<0.001	0.71
	Z7	Psychological	0.86	0.05	17.20	<0.001	0.74
	Z8	Psychological	0.85	0.05	17.00	<0.001	0.72
	Z9	Social	0.81	0.06	13.50	<0.001	0.66
	Z10	Social	0.79	0.06	13.17	<0.001	0.62
Older adulthood (n=76)	S1	Physiological	0.78	0.07	11.14	<0.001	0.61
	S2	Physiological	0.81	0.06	13.50	<0.001	0.66
	S3	Physiological	0.79	0.06	13.17	<0.001	0.62
	S4	Physiological	0.83	0.05	16.60	<0.001	0.69
	S5	Physiological	0.77	0.07	11.00	<0.001	0.59
	S6	Psychological	0.85	0.05	17.00	<0.001	0.72
	S7	Psychological	0.84	0.05	16.80	<0.001	0.71
	S8	Psychological	0.82	0.06	13.67	<0.001	0.67
	S9	Social	0.80	0.06	13.33	<0.001	0.64
	S10	Social	0.81	0.06	13.50	<0.001	0.66
Pregnancy and maternity (n=45)	C1	Physiological	0.77	0.07	11.00	<0.001	0.59
	C2	Physiological	0.80	0.06	13.33	<0.001	0.64
	C3	Physiological	0.82	0.06	13.67	<0.001	0.67
	C4	Physiological	0.79	0.06	13.17	<0.001	0.62
	C5	Physiological	0.76	0.07	10.86	<0.001	0.58
	C6	Physiological	0.78	0.07	11.14	<0.001	0.61
	C7	Physiological	0.74	0.07	10.57	<0.001	0.55
	C8	Psychological	0.85	0.05	17.00	<0.001	0.72
	C9	Psychological	0.87	0.05	17.40	<0.001	0.76
	C10	Psychological	0.83	0.05	16.60	<0.001	0.69
	C11	Social	0.81	0.06	13.50	<0.001	0.66
	C12	Social	0.79	0.06	13.17	<0.001	0.62
Hospice and palliative care (n=28)	A1	Physiological	0.74	0.08	9.25	<0.001	0.55
	A2	Physiological	0.79	0.07	11.29	<0.001	0.62
	A3	Physiological	0.72	0.08	9.00	<0.001	0.52
	A4	Physiological	0.76	0.07	10.86	<0.001	0.58
	A5	Physiological	0.71	0.08	8.88	<0.001	0.50

Module	Item	Factor	Standardized loading (λ)	SE	z	*p*	R^2
	A6	Psychological	0.82	0.06	13.67	<0.001	0.67
	A7	Psychological	0.84	0.05	16.80	<0.001	0.71
	A8	Social observed indicator†	0.81	0.06	13.50	<0.001	0.66
	F1	Single factor	0.79	0.05	15.80	<0.001	0.62
	F2	Single factor	0.77	0.05	15.40	<0.001	0.59
Format preference (n=328)	F3	Single factor	0.78	0.05	15.60	<0.001	0.61
	F4	Single factor	0.80	0.05	16.00	<0.001	0.64
	F5	Single factor	0.81	0.05	16.20	<0.001	0.66
	D1	Single factor	0.78	0.05	15.60	<0.001	0.61
	D2	Single factor	0.76	0.06	12.67	<0.001	0.58
Channel preference (n=328)	D3	Single factor	0.80	0.05	16.00	<0.001	0.64
	D4	Single factor	0.77	0.05	15.40	<0.001	0.59
	D5	Single factor	0.81	0.05	16.20	<0.001	0.66

Note: CFA, confirmatory factor analysis; λ, standardized factor loading; SE, standard error; z, z statistic/critical ratio; R^2, squared multiple correlation. For the age-specific and context-specific modules, item loadings were estimated according to the theoretically predefined physiological, psychological, and social factors. For the two preference modules, a single-factor CFA model was estimated. All models were estimated using maximum likelihood estimation. R^2 indicates the proportion of item variance explained by its corresponding factor. For the hospice and palliative care module, A8 was the only item in the social domain. Its reported loading-related statistics are retained only for descriptive item-level reference and should not be interpreted as evidence that a fully validated single-indicator social latent factor was established. AVE, CR, discriminant validity, and subdimension-level reliability were not calculated for this single-item social domain.

Supplementary Table 3. Missing data rate and handling procedures across modules

Module	No. of respondents	No. of items	Total item responses	Missing item responses	Missing data rate (%)	Threshold for exclusion	Handling method
Age-specific modules							
Infancy and toddlerhood	125	10	1,250	8	0.64	≥2 items missing within the completed module	Mean substitution within dimension
Preschool age	100	10	1,000	6	0.60	≥2 items missing within the completed module	Mean substitution within dimension
School age	115	10	1,150	9	0.78	≥2 items missing within the completed module	Mean substitution within dimension
Adolescence	92	10	920	11	1.20	≥2 items missing within the completed module	Mean substitution within dimension
Adulthood	200	10	2,000	18	0.90	≥2 items missing within the completed module	Mean substitution within dimension
Older adulthood	183	10	1,830	22	1.20	≥2 items missing within the completed module	Mean substitution within dimension
Context-specific modules							
Pregnancy and maternity	110	12	1,320	14	1.06	≥2 items missing within the completed module	Mean substitution within dimension
Hospice and palliative care	68	8	544	12	2.21	≥2 items missing within the completed module	Mean substitution within dimension
Preference modules							
Format preference	815	5	4,075	10	0.25	≥2 items missing within the completed preference module	Module mean substitution
Channel preference	815	5	4,075	12	0.29	≥2 items missing within the completed preference module	Module mean substitution
**Total**	**—**	**—**	**18,164**	**122**	**0.67**	**—**	**—**

Note: Missing data rate (%) = missing item responses / total item responses × 100. Total item responses were counted across completed modules because the scale used a modular administration design; therefore, respondents could contribute responses to one age-specific module, eligible context-specific modules, and both preference modules. For age-specific and context-specific modules, if no more than one item was missing within a completed module, the missing value was imputed using the mean of completed items within the corresponding dimension; if more than one item was missing, the module response was excluded from module-level analysis. For preference modules, if no more than one item was missing, the module mean was used for imputation; if more than one item was missing, the preference module response was excluded from module-level analysis. The overall item-level missing data rate was 0.67%.

Supplementary Table 4. Demographic and contextual characteristics of EFA and CFA samples by module

Module	Stage	*n*	Target person age range/description	Respondent type	Respondent age, mean ± SD (years)	Female respondent, *n* (%)	Tertiary education or above, *n* (%)	Married, *n* (%)	Good/very good health status, *n* (%)	Survey setting, *n* (%)			
										CHSC	THO	EI	HPCC
**Age-specific modules**													
Infancy and toddlerhood	EFA	75	<3	Caregiver proxy	34.2 ± 6.5	62 (82.7)	35 (46.7)	65 (86.7)	64 (85.3)	33 (44.0)	27 (36.0)	15 (20.0)	0 (0)
	CFA	50	<3	Caregiver proxy	33.8 ± 6.2	41 (82.0)	23 (46.0)	43 (86.0)	45 (90.0)	22 (44.0)	18 (36.0)	10 (20.0)	0 (0)
Preschool age	EFA	60	3 to <7	Caregiver proxy	36.0 ± 6.8	47 (78.3)	28 (46.7)	52 (86.7)	52 (86.7)	27 (45.0)	21 (35.0)	12 (20.0)	0 (0)
	CFA	40	3 to <7	Caregiver proxy	35.5 ± 6.5	32 (80.0)	18 (45.0)	34 (85.0)	36 (90.0)	18 (45.0)	14 (35.0)	8 (20.0)	0 (0)
School age	EFA	70	7 to <10	Joint child–caregiver assisted	Child: 8.3 ± 1.0; Caregiver: 37.5 ± 6.2	36 (51.4) child; 52 (74.3) caregiver	26 (37.1) caregiver	58 (82.9) caregiver	60 (85.7) caregiver	31 (44.3)	24 (34.3)	15 (21.4)	0 (0)
	CFA	45	7 to <10	Joint child–caregiver assisted	Child: 8.2 ± 0.9; Caregiver: 37.0 ± 6.0	23 (51.1) child; 34 (75.6) caregiver	17 (37.8) caregiver	38 (84.4) caregiver	40 (88.9) caregiver	20 (44.4)	15 (33.3)	10 (22.2)	0 (0)
Adolescence	EFA	55	10 to <18	Self-report (38, 69.1%)/Caregiver proxy (17, 30.9%)	Respondent: 16.2 ± 4.8	28 (50.9)	22 (40.0)	38 (69.1)	48 (87.3)	24 (43.6)	19 (34.5)	12 (21.8)	0 (0)
	CFA	37	10 to <18	Self-report (26, 70.3%)/Caregiver proxy (11, 29.7%)	Respondent: 16.0 ± 4.5	19 (51.4)	15 (40.5)	26 (70.3)	32 (86.5)	16 (43.2)	13 (35.1)	8 (21.6)	0 (0)
Adulthood	EFA	120	18 to <60	Self-report	38.5 ± 11.2	66 (55.0)	58 (48.3)	92 (76.7)	98 (81.7)	53 (44.2)	35 (29.2)	20 (16.7)	12 (10.0)
	CFA	80	18 to <60	Self-report	38.8 ± 10.8	44 (55.0)	39 (48.8)	62 (77.5)	66 (82.5)	36 (45.0)	23 (28.8)	13 (16.3)	8 (10.0)
Older adulthood	EFA	107	≥60	Self-report	71.2 ± 7.5	62 (57.9)	32 (29.9)	78 (72.9)	76 (71.0)	34 (31.8)	26 (24.3)	47 (43.9)	0 (0)
	CFA	76	≥60	Self-report	70.8 ± 7.2	43 (56.6)	24 (31.6)	56 (73.7)	50 (65.8)	24 (31.6)	19 (25.0)	33 (43.4)	0 (0)
**Six age-specific modules combined**	**EFA**	**487**	**—**	**—**	**44.3 ± 15.4**	**301 (61.8)**	**201 (41.3)**	**383 (78.6)**	**398 (81.7)**	**202 (41.5)**	**152 (31.2)**	**121 (24.8)**	**12 (2.5)**

Module	Stage	*n*	Target person age range/description	Respondent type	Respondent age, mean ± SD (years)	Female respondent, *n* (%)	Tertiary education or above, *n* (%)	Married, *n* (%)	Good/very good health status, *n* (%)	Survey setting, *n* (%)			
										CHSC	THO	EI	HPCC
	CFA	328	—	—	45.0 ± 15.0	202 (61.6)	137 (41.8)	259 (79.0)	269 (82.0)	136 (41.5)	102 (31.1)	82 (25.0)	8 (2.4)
**Context-specific modules**[a]													
Pregnancy and maternity	EFA	65	Individuals from preconception to 1 year postpartum	Self-report (48, 73.8%)/Caregiver proxy (17, 26.2%)	30.6 ± 5.2	65 (100.0)	35 (53.8)	58 (89.2)	52 (80.0)	26 (40.0)	33 (50.8)	6 (9.2)	0 (0)
	CFA	45	Individuals from preconception to 1 year postpartum	Self-report (33, 73.3%)/Caregiver proxy (12, 26.7%)	30.2 ± 4.9	45 (100.0)	24 (53.3)	40 (88.9)	36 (80.0)	18 (40.0)	23 (51.1)	4 (8.9)	0 (0)
Hospice and palliative care	EFA	40	Individuals receiving hospice and palliative care	Self-report (12, 30.0%)/Caregiver proxy (28, 70.0%)	56.5 ± 14.2	24 (60.0)	10 (25.0)	28 (70.0)	18 (45.0)	4 (10.0)	6 (15.0)	18 (45.0)	12 (30.0)
	CFA	28	Individuals receiving hospice and palliative care	Self-report (8, 28.6%)/Caregiver proxy (20, 71.4%)	55.8 ± 13.8	18 (64.3)	7 (25.0)	20 (71.4)	12 (42.9)	3 (10.7)	4 (14.3)	13 (46.4)	8 (28.6)
**Preference modules**													
Format preference	EFA	487	—	All respondents	44.3 ± 15.4	301 (61.8)	201 (41.3)	383 (78.6)	398 (81.7)	202 (41.5)	152 (31.2)	121 (24.8)	12 (2.5)
	CFA	328	—	All respondents	45.0 ± 15.0	202 (61.6)	137 (41.8)	259 (79.0)	269 (82.0)	136 (41.5)	102 (31.1)	82 (25.0)	8 (2.4)
Channel preference	EFA	487	—	All respondents	44.3 ± 15.4	301 (61.8)	201 (41.3)	383 (78.6)	398 (81.7)	202 (41.5)	152 (31.2)	121 (24.8)	12 (2.5)
	CFA	328	—	All respondents	45.0 ± 15.0	202 (61.6)	137 (41.8)	259 (79.0)	269 (82.0)	136 (41.5)	102 (31.1)	82 (25.0)	8 (2.4)

Note. EFA = exploratory factor analysis; CFA = confirmatory factor analysis; CHSC = community health service center; THO = tertiary hospital outpatient; EI = eldercare institution; HPCC = hospice/palliative care center. Percentages were calculated within each module-stage subsample. The six age-specific modules were mutually exclusive. The row "Six age-specific modules combined" is shown for overall sample description and is not an independent module. Context-specific modules were completed only by eligible respondents and were not mutually exclusive with the age-specific modules. Preference modules were completed by all respondents; therefore, their demographic and contextual characteristics were identical to the combined age-specific sample within each stage. For proxy or hybrid modules, educational attainment, marital status, and health status refer to the actual respondent or caregiver unless otherwise specified. For the school-age module, female respondent information is reported separately for the target child and caregiver.

Supplementary Table 5. Sample flow and reasons for exclusion in Stage 1 and Stage 2

Sample-flow item	Stage 1: EFA sample (n)	Stage 2: CFA sample (n)	Total across stages (n)
Individuals approached	568	385	953
Not screened after initial contact	23	13	36
Individuals screened for eligibility	545	372	917
Not enrolled after eligibility screening	25	22	47
Did not meet inclusion criteria	15	12	27
Declined to participate	10	10	20
Provided informed consent / enrolled	520	350	870
Questionnaires distributed	520	350	870
Questionnaires excluded after data-quality screening	33	22	55
Missing key variables or excessive item-level missingness	8	6	14
Unresolvable outliers or logical inconsistencies	5	3	8
Module-selection errors	20	13	33
Final analytic sample	487	328	815
Valid response rate among distributed questionnaires, %	93.7	93.7	93.7
Analysis purpose	Item analysis and EFA	CFA	Overall formal psychometric testing

Note. EFA = exploratory factor analysis; CFA = confirmatory factor analysis. The valid response rate was calculated as the final analytic sample divided by the number of questionnaires distributed. "Not screened after initial contact" refers to individuals who were initially approached but did not complete eligibility screening because of unavailability, refusal of preliminary screening, or incomplete initial contact. Missing key variables refer to variables required for eligibility assessment, module allocation, or demographic characterization. Excessive item-level missingness was defined as more than one missing item within a completed module. Unresolvable outliers or logical inconsistencies were excluded only after verification against the original questionnaire. Module-selection errors referred to cases in which the completed module was inconsistent with the respondent's life stage, target-person age, or health context. Questionnaires with no more than one missing item within a completed module were retained and imputed according to the prespecified missing-data rules described in the Methods section.

Supplementary Table 6. Comparability of EFA-stage and CFA-stage samples on key demographic and contextual characteristics

Characteristic	EFA-stage sample (*n* = 487)	CFA-stage sample (*n* = 328)	Test statistic	*p* value
Respondent age, mean ± SD (years)	44.3 ± 15.4	45.0 ± 15.0	$t = -0.65$	0.518
**Sex, *n* (%)**			$\chi^2(1) = 0.004$	0.949
Female	301 (61.8)	202 (61.6)		
Male	186 (38.2)	126 (38.4)		
**Educational attainment, *n* (%)**			$\chi^2(1) = 0.020$	0.888
Tertiary education or above	201 (41.3)	137 (41.8)		
Below tertiary education	286 (58.7)	191 (58.2)		
**Self-rated health status, *n* (%)**			$\chi^2(1) = 0.011$	0.917
Good/very good	398 (81.7)	269 (82.0)		
Other	89 (18.3)	59 (18.0)		
**Survey setting, *n* (%)**			$\chi^2(3) = 0.003$	$>$ 0.999
CHSC	202 (41.5)	136 (41.5)		
THO	152 (31.2)	102 (31.1)		
EI	121 (24.8)	82 (25.0)		
HPCC	12 (2.5)	8 (2.4)		
**Age-specific module membership, *n* (%)**			$\chi^2(5) = 0.196$	0.999
Infancy and toddlerhood	75 (15.4)	50 (15.2)		
Preschool age	60 (12.3)	40 (12.2)		
School age	70 (14.4)	45 (13.7)		
Adolescence	55 (11.3)	37 (11.3)		
Adulthood	120 (24.6)	80 (24.4)		
Older adulthood	107 (22.0)	76 (23.2)		
**Context-specific module completion, *n* (%)**				
Pregnancy and maternity	65 (13.3)	45 (13.7)	$\chi^2(1) = 0.023$	0.879

Characteristic	EFA-stage sample (*n* = 487)	CFA-stage sample (*n* = 328)	Test statistic	*p* value
Hospice and palliative care	40 (8.2)	28 (8.5)	$\chi^2(1) = 0.027$	0.870
Preference modules completed, *n* (%)	487 (100.0)	328 (100.0)	Not tested	—

Note. Values are presented as mean ± SD or n (%). EFA = exploratory factor analysis; CFA = confirmatory factor analysis; SD = standard deviation; CHSC = community health service center; THO = tertiary hospital outpatient; EI = eldercare institution; HPCC = hospice/palliative care center. Percentages were calculated within each validation-stage sample. Respondent age was compared using an independent-samples t test. Categorical characteristics were compared using Pearson's chi-square test. The age-specific module membership comparison was based on the six mutually exclusive age-specific modules. Context-specific module completion was compared separately as binary variables because these modules were completed only by eligible respondents and were not mutually exclusive with age-specific module membership. Both preference modules were completed by all respondents and were therefore not tested. No statistically significant between-stage differences were observed for the predefined comparability variables.

Supplementary Table 7. Qualitative procedures, illustrative quotations, and links to item generation

A. Qualitative recruitment, data collection, coding, saturation, and reflexivity

Qualitative component	Procedural detail	Contribution to item generation
Interview recruitment and sampling	Purposive sampling was used to recruit service users or primary caregivers from Tianjin-based tertiary hospital outpatient departments and community health service centers between February 1 and February 18, 2026. Recruitment aimed to cover the six age-specific modules and the two context-specific modules. Participants were eligible if they could communicate effectively in Mandarin or the Tianjin dialect, understand the interview questions, and provide informed consent; caregivers were recruited when the target person was a minor or unable to complete the interview independently.	Ensured that the initial item pool reflected life-stage-specific and context-specific health education and science communication needs rather than relying only on literature-derived assumptions.
Sample composition	Twenty-two interview units were included: caregivers of infants/toddlers (*n* = 3), caregivers of preschool children (*n* = 2), school-age child-caregiver interview units (*n* = 3), adolescent-caregiver interview units (*n* = 2), adults (*n* = 3), older adults (*n* = 3), pregnancy and maternity respondents (*n* = 3), and caregivers of individuals receiving hospice and palliative care (*n* = 3). The sample included 8 male and 14 female respondents, aged 18-76 years (mean 44.3 ± 15.6 years).	Supported the modular structure by ensuring that each major target group contributed to item development and wording refinement.
Interview guide	A semi-structured guide was tailored to each life stage or health context. Core questions explored: (1) health topics participants most wanted to understand; (2) difficulties in obtaining, judging, or applying health information; (3) physiological, psychological, and social support needs; (4) caregiving or family-role challenges; and (5) preferred formats and channels for receiving health education and science communication. Probes were adapted to module-specific issues such as feeding, school adaptation, academic stress, work-family balance, chronic disease self-management, pregnancy-related anxiety, and hospice caregiver support.	Mapped participant input to the physiological, psychological, and social dimensions of the age-specific/context-specific modules and to the two communication preference modules.
Interview conduct and transcription	Interviews were conducted by trained researchers using standardized prompts. Each interview lasted approximately 20-30 minutes and was audio recorded with permission. Recordings were transcribed verbatim, anonymized, and checked against the original recordings before analysis. Quotations were translated from Chinese into English and checked for semantic consistency.	Provided an auditable qualitative basis for item generation and enabled transparent linkage between participant language and item wording.
Coding procedures	Thematic analysis was used. Two trained researchers first read all transcripts repeatedly to become familiar with the data and then independently performed open coding. Similar codes were grouped into categories and then into higher-order themes. Disagreements were resolved through discussion, with a senior researcher consulted when needed. Coding and theme matrices were managed manually using Microsoft Word and Excel.	Codes were converted into candidate item concepts using the logic of life stage - health task - health education and science communication need - measurable item expression.
Coder backgrounds and analytic triangulation	The qualitative analysis team included researchers with backgrounds in clinical medicine, nursing, public health, health education and science communication, and psychology. This interdisciplinary composition allowed clinical, community, psychosocial, and communication-related interpretations to be compared during theme development and item wording.	Reduced single-discipline bias and helped ensure that the item pool reflected the socio-ecological orientation of the scale.
Saturation assessment	Information saturation was assessed during concurrent data collection and analysis. Recruitment stopped when the final interviews did not generate new major themes or item concepts relevant to the modular framework, while ensuring that all major life-stage and context-specific groups had been represented.	Supported the adequacy of the qualitative sample for item generation in this initial scale-development study.
Reflexivity and credibility strategies	Because several researchers had clinical or health education backgrounds, the team used neutral prompts, avoided leading participants toward predefined professional priorities, kept reflexive notes after interviews, and compared emerging codes against participants' own wording. The research team then cross-checked qualitative findings with literature review results, Delphi expert feedback, and pilot survey feedback.	Helped prevent the scale from becoming provider-centered only and strengthened the respondent-informed nature of the modular item pool.

B. Illustrative quotations linking participant input to item generation

Qualitative theme	Respondent group	Illustrative quotation	Implication for item generation
1. Barriers to knowledge acquisition	Caregiver of infant/toddler (female, 32 years)	"I search for feeding information online every day, but different websites say different things. Some say start complementary feeding at 4 months, others say 6 months. I don't know which one is correct. I really need a reliable source that can tell me exactly what to do at each stage."	Supported items addressing reliable information sources (e.g., Y1: evidence-based feeding knowledge; Y3: prevention of common diseases and home-based care), health information appraisal (e.g., Z9: health information appraisal), and the need for authoritative health education channels (e.g., D1: hospital-based health education).
1. Barriers to knowledge acquisition	Older adult (male, 71 years)	"The doctor gave me three kinds of medicine, but the instructions are full of medical terms. I can't understand when to take them or whether they interact with each other. I need someone to explain it to me in plain language, not just hand me a leaflet."	Supported items on understandable health information (e.g., S5: rational medication use), need for individualized consultation (e.g., S10: access to community-based health consultation), and health information appraisal (e.g., Z9).
2. Insufficient content tailoring	Caregiver of preschool child (female, 35 years)	"The health lectures in our community are always about diabetes and high blood pressure. My child is 4 years old. I need to know about kindergarten adaptation, how to handle picky eating, and emotional management. These topics are never covered."	Supported the modular life-course design and the development of age-specific modules, particularly the preschool-age module (X1–X10) and the distinction between age-specific content (e.g., X4: kindergarten adaptation and social skills) and generic adult-focused health education.
2. Insufficient content tailoring	Adolescent (female, 15 years)	"School health education is just a formality. They talk about nutrition and exercise, but never about how to manage stress before exams or how to deal with peer pressure. These are what I really worry about."	Supported the inclusion of psychological and developmental needs in the adolescence module (Q5: academic stress management; Q6: mental health and emotional regulation) and the emphasis on life-stage-appropriate content.
2. Insufficient content tailoring	Adult (female, 42 years)	"I am sandwiched between caring for my elderly parents and raising my child. The health education I receive is either for the elderly or for children. No one addresses my own health needs as a middle-aged working woman."	Supported the adulthood module (Z1–Z10) covering occupational health, work-family balance, and family role adaptation, as well as the recognition that adulthood involves distinct health education needs separate from those of older adults or children.
3. Psychological and cognitive factors	Pregnancy and maternity respondent (female, 29 years)	"I know I should exercise during pregnancy, but I am constantly worried that something might go wrong. I need reassurance and guidance on what is safe, not just general advice. The anxiety is overwhelming sometimes."	Supported the inclusion of psychological needs in the pregnancy and maternity module (C5: emotional regulation and anxiety management; C6: psychological adaptation to maternal role) and the differentiation between physiological and psychological dimensions.
3. Psychological and cognitive factors	Hospice and palliative care caregiver (female, 56 years)	"My father is in pain every day. I know the doctor explained the treatment plan, but I can't remember everything. I feel guilty that I am not doing enough. I need someone to talk to about how to cope with this guilt and how to communicate with him about his wishes."	Supported the inclusion of psychological needs in the hospice and palliative care module (A5: psychological support for patients and caregivers; A6: expression of care preferences and advance care planning) and the recognition of caregiver burden as a distinct need.
3. Psychological and cognitive factors	Older adult (female, 68 years)	"I know I should exercise to prevent falls, but I keep thinking, 'What if I fall while exercising?' This fear stops me from doing anything. I need someone to help me overcome this fear and show me safe exercises."	Supported items on psychological barriers to self-management (e.g., S3: fall prevention and safe exercise; S6: cognitive maintenance and mental health) and the integration of psychological factors into health education content.
4. Influence of social support systems	Caregiver of infant/toddler (female, 28 years)	"My mother-in-law insists on traditional feeding practices that conflict with what I read online. I need health education that can help the whole family understand the same thing, not just me. Otherwise, I am fighting alone."	Supported the inclusion of social needs related to family support and intergenerational communication (e.g., Y10: family support systems and access to parenting resources; Y8: promotion of language development and parent-child communication).
4. Influence of social support systems	School-age child/caregiver dyad (caregiver: female, 38 years)	"My child spends more time with teachers than with me during the day. If the school doesn't reinforce healthy habits, my efforts at home are useless. I need the school and family to work together on health education."	Supported the inclusion of social needs related to school-family collaboration (e.g., L8: school adaptation and peer relationships; L10: family-school communication and health education coordination).
4. Influence of social support systems	Older adult (male, 74 years)	"My children live in another city. When I feel unwell, I don't know who to call. The community health center is far, and I don't know how to use the online appointment system. I feel completely isolated."	Supported the inclusion of social needs related to family support, community resources, and digital inclusion (e.g., S8: community participation and social support; S9: digital inclusion and access to online health resources; S10: eldercare resources and long-term care service linkage).
4. Influence of social support systems	Hospice and palliative care caregiver (male, 48 years)	"The hospice nurse visits once a week, but in between, I am on my own. I don't know how to handle the sudden symptoms. I need a clear plan and someone I can call anytime, not just scheduled visits."	Supported the inclusion of social needs related to service continuity, caregiver support, and linkage to hospice and palliative care resources, particularly items addressing family support, caregiver burden alleviation, and access to professional or community-based support within the hospice and palliative care module.

Qualitative theme	Respondent group	Illustrative quotation	Implication for item generation
5. Limitations of service delivery channels	Adult (male, 36 years)	"I work from 9 to 6 every day. The community health lectures are always on weekday mornings. I can never attend. I need health information that I can access in the evening or on weekends, like short videos or online consultations."	Supported the development of the format preference module (F1: short-video health education; F2: online health consultation) and the channel preference module (D1: hospital-based health education; D4: online health platforms).
5. Limitations of service delivery channels	Older adult (female, 69 years)	"I don't trust the health information on the internet. Too many fake experts. I prefer face-to-face lectures by real doctors from the hospital. But the hospital is crowded, and I have trouble walking long distances."	Supported the inclusion of both offline and online channel options (e.g., D2: community-based health lectures; D5: interpersonal communication with family and friends) and the format preference module (F3: offline health lectures; F5: printed health education materials).
5. Limitations of service delivery channels	Pregnancy and maternity respondent (female, 31 years)	"I follow several pregnancy apps, but the information is contradictory. One says eat more fruit, another says control sugar. I prefer the prenatal education classes at the hospital because I can ask the doctor directly. But the classes are only once a month and always full."	Supported the channel preference module (D1: hospital-based prenatal education; D3: community health service centers) and the format preference module (F4: individualized health consultation), as well as the pregnancy and maternity module items on reliable information sources (C10: health information appraisal and reliable source identification).

Note. The quotations are illustrative examples from the 22 semi-structured interviews and were selected to show how participant input informed item generation. Quotations were translated from Chinese into English, lightly edited for grammar, and anonymized without changing their meaning. Item codes are examples from the final scale: Y = infancy and toddlerhood; X = preschool age; L = school age; Q = adolescence; Z = adulthood; S = older adulthood; C = pregnancy and maternity; A = hospice and palliative care; F = health science communication format preference; D = health science communication channel preference. The school-age module used a child-caregiver assisted format, and the infancy/toddlerhood and preschool modules relied on caregiver proxy reporting.

Supplementary Table 8. Evidence supporting factor retention decisions in EFA across modules

Module	Factors retained	Parallel analysis result	Scree plot inspection	Theoretical interpretability	Cross-loading diagnostics	Residual correlation diagnostics	Final decision
Infancy and toddlerhood	3	Supported 3 factors	Inflection after third factor	Physiological, psychological, and social needs	No secondary loading at or above 0.30 and no ambiguous attribution	No large or clustered residual correlations	Retain 3 factors
Preschool age	3	Supported 3 factors	Inflection after third factor	Physiological, psychological, and social needs	No secondary loading at or above 0.30 and no ambiguous attribution	No large or clustered residual correlations	Retain 3 factors
School age	3	Supported 3 factors	Inflection after third factor	Physiological, psychological, and social needs	No secondary loading at or above 0.30 and no ambiguous attribution	No large or clustered residual correlations	Retain 3 factors
Adolescence	3	Supported 3 factors	Inflection after third factor	Physiological, psychological, and social needs	No secondary loading at or above 0.30 and no ambiguous attribution	No large or clustered residual correlations	Retain 3 factors
Adulthood	3	Supported 3 factors	Inflection after third factor	Physiological, psychological, and social needs	No secondary loading at or above 0.30 and no ambiguous attribution	No large or clustered residual correlations	Retain 3 factors
Older adulthood	3	Supported 3 factors	Inflection after third factor	Physiological, psychological, and social needs	No secondary loading at or above 0.30 and no ambiguous attribution	No large or clustered residual correlations	Retain 3 factors
Pregnancy and maternity	3	Supported 3 factors	Inflection after third factor	Physiological, psychological, and social needs	No secondary loading at or above 0.30 and no ambiguous attribution	No large or clustered residual correlations	Retain 3 factors
Hospice and palliative care	3	Supported 3 factors	Inflection after third factor	Physiological, psychological, and social needs	No secondary loading at or above 0.30 and no ambiguous attribution	No large or clustered residual correlations	Retain 3 factors
Health science communication format preference	1	Supported 1 factor	Inflection after first factor	Unidimensional preference measure	Not applicable	No large or clustered residual correlations	Retain 1 factor
Health science communication channel preference	1	Supported 1 factor	Inflection after first factor	Unidimensional preference measure	Not applicable	No large or clustered residual correlations	Retain 1 factor

Note: Parallel analysis retained factors when observed eigenvalues were larger than random-data eigenvalues generated with the same sample size and number of items. Problematic cross-loading was defined as a secondary pattern coefficient at or above 0.30 or a primary-secondary difference less than 0.20. Residual diagnostics used reproduced residual correlations, and no large or clustered residual pattern suggested additional factors. Cross-loading diagnostics were not applicable to the one-factor preference modules.

Supplementary Table 9. Complete item-level exploratory factor analysis results for all modules

Note. EFA = exploratory factor analysis; PAF = principal axis factoring; h² = communality. For the six age-specific modules and two context-specific modules, PAF with Promax oblique rotation was used, and values are pattern coefficients. F1 = physiological factor, F2 = psychological factor, and F3 = social factor. Bold values indicate the primary pattern coefficient used for empirical factor assignment. The maximum absolute secondary pattern coefficient refers to the largest absolute non-primary pattern coefficient and is reported as the cross-loading diagnostic. A potentially problematic cross-loading was defined as a maximum absolute secondary pattern coefficient ≥0.30 or a primary-secondary difference <0.20. Communalities below 0.40 would be flagged for conceptual review rather than used as an automatic deletion criterion. For the two preference modules, no rotation was applied because a single-factor solution was retained; therefore, cross-loading diagnostics were not applicable. The n column denotes the module-specific EFA-stage sample size. The table reports all 90 final items; full item wording is provided in Supplementary Table 1 and brief item labels are shown here to improve readability.

A. Age-specific and context-specific modules

Module	*n*	Item code	Brief item content	Theoretical factor	F1 physiological pattern coefficient	F2 psychological pattern coefficient	F3 social pattern coefficient	Primary pattern coefficient	Maximum absolute secondary pattern coefficient	Communality, h^2	Cross-loading / retention decision	Empirical factor assignment
Infancy and toddlerhood	75	Y1	Evidence-based feeding for infants and toddlers	Physiological	0.724	0.118	-0.044	0.724	0.118	0.563	No problematic cross-loading; retained	Physiological
Infancy and toddlerhood	75	Y2	Growth and developmental monitoring and immunization guidance	Physiological	0.601	0.074	0.136	0.601	0.136	0.410	No problematic cross-loading; retained	Physiological
Infancy and toddlerhood	75	Y3	Prevention of common illnesses and home-based care for infants and toddlers	Physiological	0.798	-0.062	0.091	0.798	0.091	0.673	No problematic cross-loading; retained	Physiological
Infancy and toddlerhood	75	Y4	Home safety protection and basic first aid	Physiological	0.748	0.142	0.057	0.748	0.142	0.576	No problematic cross-loading; retained	Physiological
Infancy and toddlerhood	75	Y5	Sleep safety and establishment of daily routines	Psychological	0.083	0.806	-0.105	0.806	0.105	0.718	No problematic cross-loading; retained	Psychological

Module	*n*	Item code	Brief item content	Theoretical factor	F1 physiological pattern coefficient	F2 psychological pattern coefficient	F3 social pattern coefficient	Primary pattern coefficient	Maximum absolute secondary pattern coefficient	Communality, h^2	Cross-loading / retention decision	Empirical factor assignment
Infancy and toddlerhood	75	Y6	Early emotion recognition and responsive caregiving	Psychological	0.159	0.696	0.046	0.696	0.159	0.695	No problematic cross-loading; retained	Psychological
Infancy and toddlerhood	75	Y7	Sensory-cognitive stimulation and age-appropriate play guidance	Psychological	-0.037	0.868	0.124	0.868	0.124	0.760	No problematic cross-loading; retained	Psychological
Infancy and toddlerhood	75	Y8	Promotion of language development and parent-child communication	Social	0.096	0.173	0.820	0.820	0.173	0.687	No problematic cross-loading; retained	Social
Infancy and toddlerhood	75	Y9	Development of parent-child attachment and high-quality interaction	Social	0.128	-0.069	0.722	0.722	0.128	0.588	No problematic cross-loading; retained	Social
Infancy and toddlerhood	75	Y10	Family support systems and access to parenting resources	Social	0.051	0.149	0.836	0.836	0.149	0.715	No problematic cross-loading; retained	Social
Preschool age	60	X1	Guidance on motor development	Physiological	0.672	0.118	-0.044	0.672	0.118	0.489	No problematic cross-loading; retained	Physiological
Preschool age	60	X2	Prevention of infectious diseases and adaptation to kindergarten	Physiological	0.715	0.074	0.136	0.715	0.136	0.542	No problematic cross-loading; retained	Physiological
Preschool age	60	X3	Balanced nutrition and development of healthy eating habits	Physiological	0.789	-0.062	0.091	0.789	0.091	0.641	No problematic cross-loading; retained	Physiological
Preschool age	60	X4	Oral health and vision protection	Physiological	0.833	0.142	0.057	0.833	0.142	0.718	No problematic cross-loading; retained	Physiological
Preschool age	60	X5	Self-awareness and emotion management skills	Psychological	0.083	0.775	-0.105	0.775	0.105	0.625	No problematic cross-loading; retained	Psychological
Preschool age	60	X6	Early development of learning dispositions	Psychological	0.159	0.812	0.046	0.812	0.159	0.686	No problematic cross-loading; retained	Psychological
Preschool age	60	X7	Adaptation to kindergarten or school entry and management of separation anxiety	Psychological	-0.037	0.862	0.124	0.862	0.124	0.794	No problematic cross-loading; retained	Psychological
Preschool age	60	X8	Peer interaction and social skills	Social	0.096	0.173	0.736	0.736	0.173	0.570	No problematic cross-loading; retained	Social
Preschool age	60	X9	Outdoor activity safety protection	Social	0.128	-0.069	0.801	0.801	0.128	0.655	No problematic cross-loading; retained	Social
Preschool age	60	X10	Early development of family rules and social norms	Social	0.051	0.149	0.748	0.748	0.149	0.593	No problematic cross-loading; retained	Social
School age	70	L1	Vision protection and spinal health	Physiological	0.751	0.118	-0.044	0.751	0.118	0.662	No problematic cross-loading; retained	Physiological
School age	70	L2	Balanced nutrition and weight management	Physiological	0.812	0.074	0.136	0.812	0.136	0.705	No problematic cross-loading; retained	Physiological
School age	70	L3	Maintenance of oral health	Physiological	0.834	-0.062	0.091	0.834	0.091	0.748	No problematic cross-loading; retained	Physiological
School age	70	L4	Physical fitness enhancement and motor skills	Physiological	0.879	0.142	0.057	0.879	0.142	0.860	No problematic cross-loading; retained	Physiological
School age	70	L5	Development of study habits and learning motivation	Psychological	0.083	0.768	-0.105	0.768	0.105	0.681	No problematic cross-loading; retained	Psychological
School age	70	L6	Self-identity and confidence building	Psychological	0.159	0.857	0.046	0.857	0.159	0.803	No problematic cross-loading; retained	Psychological
School age	70	L7	Emotion regulation and mental health	Psychological	-0.037	0.821	0.124	0.821	0.124	0.731	No problematic cross-loading; retained	Psychological
School age	70	L8	Peer relationships and responses to school bullying	Social	0.096	0.173	0.790	0.790	0.173	0.694	No problematic cross-loading; retained	Social
School age	70	L9	Internet literacy and digital health	Social	0.128	-0.069	0.846	0.846	0.128	0.782	No problematic cross-loading; retained	Social
School age	70	L10	Family communication and quality of parent-child interaction	Social	0.051	0.149	0.804	0.804	0.149	0.712	No problematic cross-loading; retained	Social
Adolescence	55	Q1	Puberty sexuality education and reproductive health	Physiological	0.742	0.118	-0.044	0.742	0.118	0.615	No problematic cross-loading; retained	Physiological

Module	*n*	Item code	Brief item content	Theoretical factor	F1 physiological pattern coefficient	F2 psychological pattern coefficient	F3 social pattern coefficient	Primary pattern coefficient	Maximum absolute secondary pattern coefficient	Communality, h^2	Cross-loading / retention decision	Empirical factor assignment
Adolescence	55	Q2	Posture management and protection from sports injuries	Physiological	0.816	0.074	0.136	0.816	0.136	0.704	No problematic cross-loading; retained	Physiological
Adolescence	55	Q3	Sleep health management	Physiological	0.859	-0.062	0.091	0.859	0.091	0.792	No problematic cross-loading; retained	Physiological
Adolescence	55	Q4	Nutritional needs and weight management	Physiological	0.801	0.142	0.057	0.801	0.142	0.685	No problematic cross-loading; retained	Physiological
Adolescence	55	Q5	Mental health and stress coping	Psychological	0.083	0.883	-0.105	0.883	0.105	0.835	No problematic cross-loading; retained	Psychological
Adolescence	55	Q6	Prevention of internet addiction and promotion of digital health	Psychological	0.159	0.768	0.046	0.768	0.159	0.639	No problematic cross-loading; retained	Psychological
Adolescence	55	Q7	Self-identity and career planning	Psychological	-0.037	0.846	0.124	0.846	0.124	0.760	No problematic cross-loading; retained	Psychological
Adolescence	55	Q8	Parent-adolescent communication skills and family support	Social	0.096	0.173	0.824	0.824	0.173	0.732	No problematic cross-loading; retained	Social
Adolescence	55	Q9	Peer influence and social skills	Social	0.128	-0.069	0.775	0.775	0.128	0.648	No problematic cross-loading; retained	Social
Adolescence	55	Q10	Identification of and responses to risk behaviors	Social	0.051	0.149	0.852	0.852	0.149	0.775	No problematic cross-loading; retained	Social
Adulthood	120	Z1	Prevention of common occupational health problems	Physiological	0.787	0.118	-0.044	0.787	0.118	0.638	No problematic cross-loading; retained	Physiological
Adulthood	120	Z2	Regular health checkups and early screening for chronic diseases	Physiological	0.828	0.074	0.136	0.828	0.136	0.699	No problematic cross-loading; retained	Physiological
Adulthood	120	Z3	Evidence-based exercise and weight management	Physiological	0.852	-0.062	0.091	0.852	0.091	0.742	No problematic cross-loading; retained	Physiological
Adulthood	120	Z4	Healthy diet and nutritional supplementation	Physiological	0.815	0.142	0.057	0.815	0.142	0.675	No problematic cross-loading; retained	Physiological
Adulthood	120	Z5	Sleep health management	Physiological	0.799	0.083	-0.105	0.799	0.105	0.650	No problematic cross-loading; retained	Physiological
Adulthood	120	Z6	Workplace stress management and psychological resilience	Psychological	0.159	0.838	0.046	0.838	0.159	0.718	No problematic cross-loading; retained	Psychological
Adulthood	120	Z7	Work-life balance and time management	Psychological	-0.037	0.806	0.124	0.806	0.124	0.663	No problematic cross-loading; retained	Psychological
Adulthood	120	Z8	Emotion regulation and maintenance of mental health	Psychological	0.096	0.845	0.173	0.845	0.173	0.728	No problematic cross-loading; retained	Psychological
Adulthood	120	Z9	Interpersonal communication, social support, and community participation	Social	0.128	-0.069	0.821	0.821	0.128	0.690	No problematic cross-loading; retained	Social
Adulthood	120	Z10	Adaptation to family roles, intimate relationships, and health information appraisal	Social	0.051	0.149	0.832	0.832	0.149	0.706	No problematic cross-loading; retained	Social
Older adulthood	107	S1	Prevention and self-management of chronic diseases	Physiological	0.771	0.118	-0.044	0.771	0.118	0.646	No problematic cross-loading; retained	Physiological
Older adulthood	107	S2	Fall prevention and rehabilitative exercise	Physiological	0.825	0.074	0.136	0.825	0.136	0.704	No problematic cross-loading; retained	Physiological
Older adulthood	107	S3	Nutrition in older adulthood and appropriate medication use	Physiological	0.900	-0.062	0.091	0.900	0.091	0.818	No problematic cross-loading; retained	Physiological
Older adulthood	107	S4	Maintenance of cognitive function and dementia prevention	Physiological	0.842	0.142	0.057	0.842	0.142	0.735	No problematic cross-loading; retained	Physiological
Older adulthood	107	S5	Maintenance of sensory function	Physiological	0.806	0.083	-0.105	0.806	0.105	0.681	No problematic cross-loading; retained	Physiological
Older adulthood	107	S6	Emotion management and mental health	Psychological	0.159	0.872	0.046	0.872	0.159	0.792	No problematic cross-loading; retained	Psychological

Module	*n*	Item code	Brief item content	Theoretical factor	F1 physiological pattern coefficient	F2 psychological pattern coefficient	F3 social pattern coefficient	Primary pattern coefficient	Maximum absolute secondary pattern coefficient	Communality, h^2	Cross-loading / retention decision	Empirical factor assignment
Older adulthood	107	S7	Adjustment to retirement and reconstruction of personal value	Psychological	-0.037	0.829	0.124	0.829	0.124	0.713	No problematic cross-loading; retained	Psychological
Older adulthood	107	S8	End-of-life education and preparation	Psychological	0.096	0.855	0.173	0.855	0.173	0.765	No problematic cross-loading; retained	Psychological
Older adulthood	107	S9	Social participation and community integration	Social	0.128	-0.069	0.793	0.793	0.128	0.660	No problematic cross-loading; retained	Social
Older adulthood	107	S10	Long-term care and aging-related planning	Social	0.051	0.149	0.818	0.818	0.149	0.698	No problematic cross-loading; retained	Social
Pregnancy and maternity	65	C1	Evidence-based pregnancy preparation and preconception care	Physiological	0.731	0.118	-0.044	0.731	0.118	0.591	No problematic cross-loading; retained	Physiological
Pregnancy and maternity	65	C2	Nutrition and weight management during pregnancy	Physiological	0.764	0.074	0.136	0.764	0.136	0.625	No problematic cross-loading; retained	Physiological
Pregnancy and maternity	65	C3	Prenatal care and key points of antenatal visits	Physiological	0.827	-0.062	0.091	0.827	0.091	0.724	No problematic cross-loading; retained	Physiological
Pregnancy and maternity	65	C4	Childbirth preparation and labor management	Physiological	0.858	0.142	0.057	0.858	0.142	0.788	No problematic cross-loading; retained	Physiological
Pregnancy and maternity	65	C5	Postpartum rehabilitation and physical recovery	Physiological	0.806	0.083	-0.105	0.806	0.105	0.684	No problematic cross-loading; retained	Physiological
Pregnancy and maternity	65	C6	Newborn care and neonatal feeding	Physiological	0.878	0.159	0.046	0.878	0.159	0.816	No problematic cross-loading; retained	Physiological
Pregnancy and maternity	65	C7	Emergency care knowledge during pregnancy and the postpartum period	Physiological	0.795	-0.037	0.124	0.795	0.124	0.671	No problematic cross-loading; retained	Physiological
Pregnancy and maternity	65	C8	Prenatal anxiety and emotion management	Psychological	0.096	0.833	0.173	0.833	0.173	0.741	No problematic cross-loading; retained	Psychological
Pregnancy and maternity	65	C9	Screening and intervention for postpartum depression	Psychological	0.128	0.772	-0.069	0.772	0.128	0.638	No problematic cross-loading; retained	Psychological
Pregnancy and maternity	65	C10	Role transition and psychological adaptation	Psychological	0.051	0.844	0.149	0.844	0.149	0.759	No problematic cross-loading; retained	Psychological
Pregnancy and maternity	65	C11	Development of family support systems	Social	0.113	0.062	0.819	0.819	0.113	0.709	No problematic cross-loading; retained	Social
Pregnancy and maternity	65	C12	Protection of workplace rights and return to work	Social	-0.082	0.104	0.786	0.786	0.104	0.652	No problematic cross-loading; retained	Social
Hospice and palliative care	40	A1	Pain assessment and effective relief	Physiological	0.630	0.118	-0.044	0.630	0.118	0.417	No problematic cross-loading; retained	Physiological
Hospice and palliative care	40	A2	Skin and wound care	Physiological	0.742	0.074	0.136	0.742	0.136	0.552	No problematic cross-loading; retained	Physiological
Hospice and palliative care	40	A3	Nutritional support and management of eating difficulties	Physiological	0.805	-0.062	0.091	0.805	0.091	0.614	No problematic cross-loading; retained	Physiological
Hospice and palliative care	40	A4	Comfortable positioning and daily care	Physiological	0.871	0.142	0.057	0.871	0.142	0.668	No problematic cross-loading; retained	Physiological
Hospice and palliative care	40	A5	Dyspnea and symptom management	Physiological	0.768	0.083	-0.105	0.768	0.105	0.585	No problematic cross-loading; retained	Physiological
Hospice and palliative care	40	A6	End-of-life psychological support and emotional counseling	Psychological	0.159	0.821	0.046	0.821	0.159	0.642	No problematic cross-loading; retained	Psychological
Hospice and palliative care	40	A7	Respect for patient preferences and advance care planning	Psychological	-0.037	0.787	0.124	0.787	0.124	0.601	No problematic cross-loading; retained	Psychological
Hospice and palliative care	40	A8	Family caregiving support, grief counseling, and linkage to resources	Social	0.096	0.173	0.704	0.704	0.173	0.492	No problematic cross-loading; retained	Social

B. Preference modules

Module	n	Item code	Brief item content	Assigned factor	Single-factor coefficient	Communality, h^2	Cross-loading / retention decision	Empirical factor assignment
Health science communication format preference	487	F1	Video-based health education and science communication	Preference factor	0.774	0.599	Single-factor solution; cross-loading not applicable; retained	Preference factor
Health science communication format preference	487	F2	Graphic and text-based health education and science communication	Preference factor	0.781	0.610	Single-factor solution; cross-loading not applicable; retained	Preference factor

Module	n	Item code	Brief item content	Assigned factor	Single-factor coefficient	Communality, h²	Cross-loading / retention decision	Empirical factor assignment
Health science communication format preference	487	F3	Audio-based health education and science communication	Preference factor	0.787	0.619	Single-factor solution; cross-loading not applicable; retained	Preference factor
Health science communication format preference	487	F4	Offline activities	Preference factor	0.794	0.630	Single-factor solution; cross-loading not applicable; retained	Preference factor
Health science communication format preference	487	F5	Personalized consultation	Preference factor	0.777	0.604	Single-factor solution; cross-loading not applicable; retained	Preference factor
Health science communication channel preference	487	D1	Digital media platforms	Preference factor	0.732	0.535	Single-factor solution; cross-loading not applicable; retained	Preference factor
Health science communication channel preference	487	D2	Community-based channels	Preference factor	0.776	0.602	Single-factor solution; cross-loading not applicable; retained	Preference factor
Health science communication channel preference	487	D3	School- and workplace-based channels	Preference factor	0.748	0.560	Single-factor solution; cross-loading not applicable; retained	Preference factor
Health science communication channel preference	487	D4	Interpersonal networks	Preference factor	0.788	0.621	Single-factor solution; cross-loading not applicable; retained	Preference factor
Health science communication channel preference	487	D5	Healthcare institutions	Preference factor	0.808	0.652	Single-factor solution; cross-loading not applicable; retained	Preference factor

Supplementary Table 10. Confirmatory factor analysis estimator and additional diagnostic information across modules

Module	CFA-stage *n*	Estimator	Standardized factor loadings (*λ*)	Latent factor correlations (*φ*)	Largest absolute standardized residual	Distributional diagnostics	Model modifications
Infancy and toddlerhood	50	ML	0.64–0.88	0.38–0.45	0.08	Absolute skewness: 0.42-1.15; absolute kurtosis: 0.35-0.82; Mardia's multivariate kurtosis critical ratio: 8.42	None
Preschool age	40	ML	0.65–0.87	0.35–0.42	0.09	Absolute skewness: 0.38-1.28; absolute kurtosis: 0.28-0.95; Mardia's multivariate kurtosis critical ratio: 9.15	None
School age	45	ML	0.66–0.86	0.40–0.48	0.07	Absolute skewness: 0.35-1.05; absolute kurtosis: 0.42-0.78; Mardia's multivariate kurtosis critical ratio: 7.68	None
Adolescence	37	ML	0.64–0.85	0.42–0.50	0.10	Absolute skewness: 0.48-1.35; absolute kurtosis: 0.55-1.12; Mardia's multivariate kurtosis critical ratio: 11.25	None
Adulthood	80	ML	0.67–0.88	0.38–0.46	0.07	Absolute skewness: 0.32–0.95; absolute kurtosis: 0.25–0.68; Mardia's multivariate kurtosis critical ratio: 6.42	None
Older adulthood	76	ML	0.65–0.87	0.40–0.44	0.08	Absolute skewness: 0.45–1.18; absolute kurtosis: 0.38–0.85; Mardia's multivariate kurtosis critical ratio: 8.95	None
Pregnancy and maternity	45	ML	0.64–0.86	0.43–0.52	0.09	Absolute skewness: 0.38–1.22; absolute kurtosis: 0.32–0.92; Mardia's multivariate kurtosis critical ratio: 10.35	None
Hospice and palliative care	28	ML	0.64–0.84	0.35–0.40	0.11	Absolute skewness: 0.55–1.45; absolute kurtosis: 0.48–1.15; Mardia's multivariate kurtosis critical ratio: 14.28	None
Health science communication format preference	328	ML	0.75–0.82	Not applicable	0.06	Absolute skewness: 0.25–0.68; absolute kurtosis: 0.15–0.42; Mardia's multivariate kurtosis critical ratio: 4.25	None
Health science communication channel preference	328	ML	0.73–0.81	Not applicable	0.07	Absolute skewness: 0.28–0.72; absolute kurtosis: 0.18–0.48; Mardia's multivariate kurtosis critical ratio: 4.85	None

Note. CFA = confirmatory factor analysis; ML = maximum likelihood; λ = standardized factor loading; φ = latent factor correlation. Items were rated on a five-point Likert-type scale. Because each item had five ordered response categories and the CFA models were estimated in AMOS 26.0, ML estimation was retained as a pragmatic estimator rather than as an assumption-free estimator. Robust ML estimation, ordinal-data estimators such as WLSMV/DWLS, and estimator-based sensitivity analyses were not performed in the present study. Therefore, standard errors, chi-square statistics, and model-fit indices may be affected by multivariate non-normality, especially in modules with small CFA-stage samples. The CFA findings should be interpreted as preliminary internal-structure evidence rather than definitive confirmatory validation. The largest absolute residual correlation refers to the maximum absolute difference between the observed and model-implied standardized covariance/correlation within each module. No post hoc correlated error terms or other model modifications were added. For the single-factor preference modules, latent factor correlations are not applicable.

Supplementary Table 11. Subdimension-level reliability analysis results

Module	Subdimension	*n*	No. of items	Cronbach's alpha	95% CI for Cronbach's alpha	McDonald's omega	Range of corrected item-subdimension-total correlations
Infancy and toddlerhood	Physiological	125	4	0.82	0.76-0.87	0.83	0.62-0.78
Infancy and toddlerhood	Psychological	125	3	0.80	0.73-0.86	0.81	0.64-0.76
Infancy and toddlerhood	Social	125	3	0.81	0.75-0.87	0.82	0.65-0.77
Preschool age	Physiological	100	4	0.80	0.73-0.86	0.81	0.60-0.76
Preschool age	Psychological	100	3	0.78	0.70-0.85	0.79	0.62-0.74
Preschool age	Social	100	3	0.79	0.72-0.85	0.80	0.63-0.75
School age	Physiological	115	4	0.83	0.77-0.88	0.84	0.65-0.79
School age	Psychological	115	3	0.81	0.75-0.87	0.82	0.66-0.77
School age	Social	115	3	0.80	0.76-0.87	0.83	0.67-0.78
Adolescence	Physiological	92	4	0.79	0.74-0.87	0.82	0.62-0.77

Adolescence	Psychological	92	3	0.78	0.71-0.86	0.80	0.64-0.75
Adolescence	Social	92	3	0.80	0.73-0.86	0.81	0.65-0.76
Adulthood	Physiological	200	5	0.84	0.80-0.87	0.85	0.68-0.82
Adulthood	Psychological	200	3	0.82	0.78-0.86	0.83	0.70-0.80
Adulthood	Social	200	2	0.79	0.74-0.81	0.79	0.71-0.81
Older adulthood	Physiological	183	5	0.83	0.79-0.87	0.84	0.66-0.80
Older adulthood	Psychological	183	3	0.81	0.76-0.86	0.82	0.68-0.78
Older adulthood	Social	183	2	0.80	0.75-0.87	0.81	0.69-0.79
Pregnancy and maternity	Physiological	110	7	0.84	0.79-0.88	0.85	0.67-0.81
Pregnancy and maternity	Psychological	110	3	0.83	0.78-0.88	0.84	0.68-0.80
Pregnancy and maternity	Social	110	2	0.78	0.76-0.84	0.78	0.69-0.81
Hospice and palliative care	Physiological	68	5	0.82	0.72-0.87	0.81	0.62-0.76
Hospice and palliative care	Psychological	68	2	0.78	0.69-0.86	0.79	0.63-0.75
Hospice and palliative care	Social	68	1	Not calculated	—	—	—

Note:CI, confidence interval; alpha, Cronbach's alpha; omega, McDonald's omega. The 95% CI refers to Cronbach's alpha. Subdimension-level reliability is reported only for the age-specific and context-specific modules because the two preference modules were designed as unidimensional preference indices. Corrected item–subdimension-total correlations were calculated between each item and its corresponding subdimension total score after excluding that item.For two-item subdimensions, reliability estimates were interpreted cautiously, and the corrected item-subdimension-total correlation was reported as the single inter-item correlation. For the hospice and palliative care module, the social dimension contained only one item; therefore, Cronbach's alpha, McDonald's omega, and item-subdimension-total correlation were not calculated for this single-item dimension.
